# Deciphering highly similar multigene family transcripts from Iso-Seq data with IsoCon

**DOI:** 10.1038/s41467-018-06910-x

**Published:** 2018-11-02

**Authors:** Kristoffer Sahlin, Marta Tomaszkiewicz, Kateryna D. Makova, Paul Medvedev

**Affiliations:** 1 Department of Computer Science and Engineering, Pennsylvania State University, University Park, PA 16802 USA; 2 Department of Biology, Pennsylvania State University, University Park, PA 16802 USA; 3 Center for Medical Genomics, Huck Institutes of the Life Sciences, Pennsylvania State University, University Park, PA 16802 USA; 4 Center for Computational Biology and Bioinformatics, Huck Institutes of the Life Sciences, Pennsylvania State University, University Park, PA 16802 USA; 5 Department of Biochemistry and Molecular Biology, Pennsylvania State University, University Park, PA 16802 USA

## Abstract

A significant portion of genes in vertebrate genomes belongs to multigene families, with each family containing several gene copies whose presence/absence, as well as isoform structure, can be highly variable across individuals. Existing de novo techniques for assaying the sequences of such highly-similar gene families fall short of reconstructing end-to-end transcripts with nucleotide-level precision or assigning alternatively spliced transcripts to their respective gene copies. We present IsoCon, a high-precision method using long PacBio Iso-Seq reads to tackle this challenge. We apply IsoCon to nine Y chromosome ampliconic gene families and show that it outperforms existing methods on both experimental and simulated data. IsoCon has allowed us to detect an unprecedented number of novel isoforms and has opened the door for unraveling the structure of many multigene families and gaining a deeper understanding of genome evolution and human diseases.

## Abstract (web summary)

Transcripts from highly-similar multigene families are challenging to decipher. Here, the authors develop IsoCon, a tool for detecting and reconstructing isoforms from multigene families by analyzing long PacBio Iso-Seq reads.

## Introduction

A significant portion of genes in the human genome belongs to multigene families, with each family containing several gene copies that have arisen via duplication, i.e. duplicate gene copies^1–6^. Many of these duplicate genes have been associated with important human phenotypes, including a number of diseases^7–9^. In some of such cases, individual gene copies play different roles in disease etiology^10^. However, the annotation of multigene families remains incomplete even in the latest human assembly, especially due to unresolved segmental duplications with high sequence identity^11,12^. Duplicate gene copies from the same family vary in sequence identity, with some of them being identical to each other. Additionally, copy numbers within families frequently differ among individuals^1–3^. Furthermore, an estimated >90% of all multi-exon genes are alternatively spliced in humans^13,14^, and different duplicate gene copies can vary in alternatively spliced forms (i.e. isoforms) produced.

These features make deciphering the end-to-end transcript sequences from duplicate genes and their various transcript isoforms a major challenge. The copy number of multigene families can be assayed using microarrays^2^, quantitative polymerase chain reaction (PCR)^15^, droplet digital PCR^16^, or DNA sequencing using Nanostring Technologies^8^ or Illumina platforms^3^. Sequences of individual exons that are only a few hundred nucleotides long can be obtained from individual reads of Illumina DNA or RNA-seq data^17^; however, the repetitive nature of duplicate gene copies complicates their de novo assemblies, and Illumina reads are often unable to phase variants across the length of the full transcript and have low recall rate in assemblies of genes with multiple isoforms^18,19^. Long Pacific Biosciences (PacBio) reads from the Iso-Seq protocol hold the potential to overcome this challenge by sequencing many transcripts end to end. This approach has been successfully applied to reveal several complex isoform structures resulting from alternative splicing events in, e.g., humans, plants, and fungi^18,20,21^. None of these studies has simultaneously tackled the problems of deciphering isoform structure and of determining which gene copies they originated from.

While PacBio error rates have decreased, many errors remain hard to correct and remain a significant problem for downstream analyses of Iso-Seq data^19,22,23^. This is especially the case for transcripts from gene families with high sequence identity, where teasing out errors from true variants is difficult. The use of a reference genome^24–28^ for correction is not effective in such situations, where the variability of gene copies might not be reliably captured by the reference. ICE^18^, a part of PacBio’s bioinformatic pipeline to process Iso-Seq data, is the standard tool employed to correct sequencing errors without using the reference. Though ICE has been utilized in several projects^29,30^, it has been shown to generate a large number of redundant transcripts^18,21,31^. Moreover, ICE “is not currently customized to work for differentiating highly complex gene families from polyploid species where differences are mostly SNP-based.”^32^. An alternate approach—to use Illumina reads to correct errors in PacBio reads^25,26,28,33^—is similarly unable to correct most errors (as we demonstrate in this paper) and is also biased by low Illumina read depth in GC-rich regions^34^.

Some approaches were proposed to error correct PacBio reads from transcripts with high sequence identity, but none are broadly applicable to determining the sequences from high-sequence-identity multigene families without relying on the reference genome. Classification^35^ or construction^36^ of allele-specific transcripts with Iso-Seq have been described, but these approaches require a reference and can only separate two alleles of a single gene. Genotyping approaches for multigene families have also been proposed^37,38^, but they require prior knowledge of the isoform sequences. A de novo approach for clustering highly similar isoforms is described in ref. ^39^, but no implementation is provided. The problem is also related to that of viral phasing^40^, but the techniques developed there are not directly applicable to multigene families.

Another consideration is the relatively high cost of PacBio. The number of reads required to recover gene families whose expression is dwarfed by super-prevalent mRNA classes can be prohibitive. A targeted sequencing approach can be effective at reducing the necessary amount of sequencing, where RT-PCR primer pairs are designed to pull out transcripts of the gene family of interest^41^. This approach results in sequencing depths high enough to capture most transcripts and perform downstream error correction.

To address these limitations, we develop IsoCon, a de novo algorithm for error-correcting and removing redundancy of PacBio circular consensus sequence (CCS) reads generated from targeted sequencing with the Iso-Seq protocol. Our algorithm allows one to decipher isoform sequences down to the nucleotide level and hypothesize how they are assigned to individual, highly similar gene copies of multigene families. IsoCon uses a cautiously iterative process to correct obvious errors, without overcorrecting rare variants. Its statistical framework is designed to leverage the power of long reads to link variants across the transcript. Furthermore, IsoCon statistically integrates the large variability in read quality, which tends to decrease as the transcript gets longer. Using simulated data, we demonstrate that IsoCon has substantially higher precision and recall than ICE^18^ across a wide range of sequencing depths, as well as of transcript lengths, similarities and abundance levels.

We apply IsoCon to the study of Y chromosome ampliconic gene families, where the inability to study separate gene copies and their respective transcripts has limited our understanding of the evolution of the primate Y chromosome and the causes of male infertility disorders for which these genes are crucial^42–45^. Y chromosome ampliconic gene families represent a particularly interesting and challenging case to decipher, because each of them contains several nearly identical (up to 99.99%) copies^46,47^ with a potentially varying number of isoforms. We use a targeted design to isolate and sequence transcripts from all nine Y chromosome ampliconic gene families from the testes of two men. Our validation shows that IsoCon drastically increases precision compared to both ICE and Illumina-based error correction with proovread^48^ and has significantly higher recall than ICE. We show that IsoCon can detect rare transcripts that differ by as little as one base pair from dominant isoforms that have two orders of magnitude higher abundance. Using IsoCon’s predicted transcripts, we are able to capture an unprecedented number of isoforms that are absent from existing databases. We are further able to separate transcripts into putative gene copies and derive copy-specific exon sequences and splice variants.

To demonstrate the broader applicability of IsoCon, we also run it on a publically available dataset of targeted Iso-Seq sequencing of the *FMR1* gene^49^. *FMR1* is a member of the fragile X-related gene family and is responsible for developing both fragile X syndrome and Fragile X-associated Tremor/Ataxia Syndrome, an adult onset neurodegenerative disorder. *FMR1* undergoes extensive alternative splicing which has been the subject of several studies^49^. Using IsoCon, we are able to recover more isoforms than ICE and find novel candidate splice junctions. Our findings suggest the difference in the number of isoforms between carriers and controls is not as large as was previously reported in ref. ^49^.

## Results

### Simulated data

We generated synthetic gene families using three reference genes as the starting sequence for our simulation: *TSPY*, *HSFY*, and *DAZ*. We chose these because they reflect the spectrum of length, exon number, and complexity, that is characteristic of Y ampliconic gene families (Table 1). *DAZ* is the hardest case, since it has a highly repetitive exon structure^50^. Gene length is also important, since longer transcripts result in fewer passes of the polymerase during sequencing and, hence, a higher error rate of CCS reads. We simulated coverage levels in a range consistent with what we observed in real data.

**Table 1:** Simulated data information. Gene sequences (taken from the corresponding reference gene in Ensembl) and their corresponding PacBio CCS error rates used for simulation. We simulated multigene families by using these gene sequences at the root. We refer to the resulting gene families by using the name of the reference gene, sometimes dropping the last modifier (i.e*. TSPY* instead of *TSPY13P*)

Reference gene	Sum of exon lengths (nt)	Number of exons	Overall simulation error rate (%)	Median errors per simulated transcript	Median no. of passes per simulated transcript
*TSPY13P*	914	6	0.5	2	18
*HSFY2*	2668	6	2.6	41	6
*DAZ2*	5904	28	6.1	276	2

**Fig. 1:**
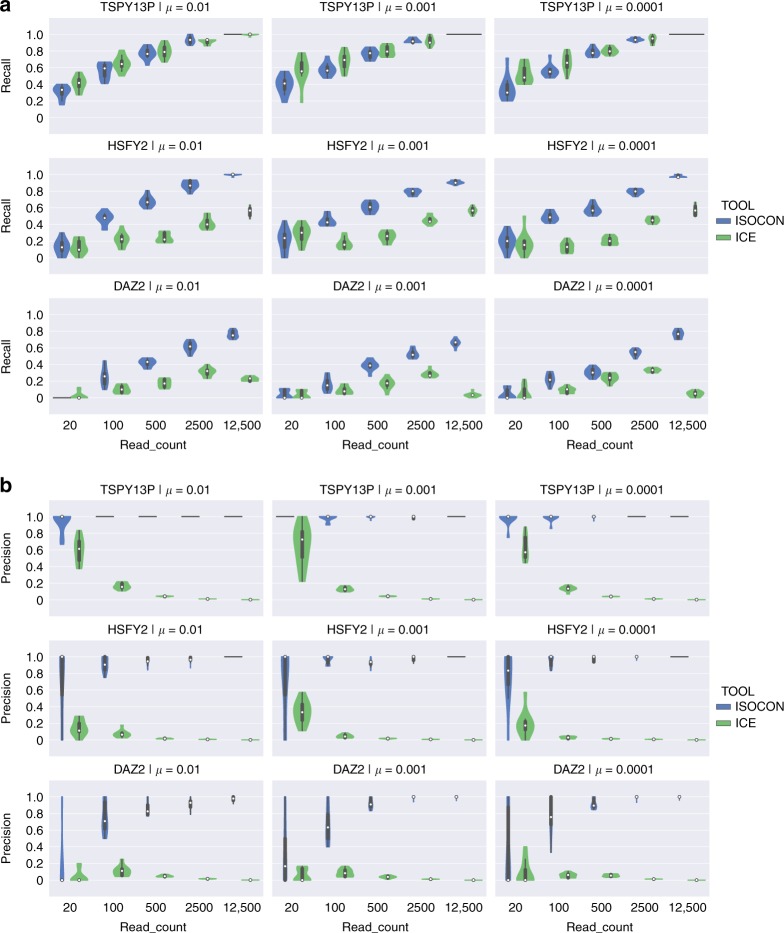
Recall and precision for transcripts with different exon structure and unequal abundance rates. Violin plots showing the recall (**a**) and precision (**b**) of IsoCon and ICE. In each panel, the rows correspond to different families and, hence, different error rates. The shortest gene family (*TSPY*, labeled by the name of the reference gene copy used to generate it, *TSPY13P*), with correspondingly lower read error rates, is shown in the top panel rows while the longest gene family (*DAZ*, labeled as *DAZ2*), with correspondingly higher read error rates, is shown in the bottom rows. The columns correspond to different mutation rates (*μ*) used in simulating the gene copies (see Supplementary Note 2). A lower mutation rate implies more similar gene copies. Each plot shows results for a total of 30 isoforms with abundances randomly assigned and ranging from 0.1% to 15%. Within each plot, the *x*-axis corresponds to the number of simulated reads, while the *y*-axis shows the recall/precision of the methods. Each violin is generated using 10 simulated sequencing replicates. The white dot shows median, the thick black line is the interquartile range (middle 50%), the thinner black line is the 95% confidence interval, and the colored area is the density plot. We note that the density plot is cut at the most extreme data points

Our main simulation focused on two scenarios. The first one (Fig. 1) reflects a typical biological scenario. For each of the three gene families, we simulated several gene copies and, for each copy, we simulated various isoforms by skipping different exons. There were a total of 30 simulated isoforms per family, with absolute abundances randomly assigned from the values 2^*i*^, *i*∈[1,8], leading to a relative abundance from 0.1% to 15%. We generate three such datasets by varying the mutation rate used to generate duplicate gene copies. (Note that here for simplicity we model mutation only, although other processes, e.g., gene conversion, are known to influence evolution of duplicate gene copies^51^.) The second simulation (Supplementary Fig. 1) is similar to the first, but, in order to tease out the effect of mutation from that of exon skipping, we do not simulate isoforms. For each gene family, there were a total of eight gene copies and eight transcripts (one per gene copy) simulated, with varying sequence identity (Supplementary Fig. 2) and with relative abundances ranging from 0.4% to 50%. We also repeated these two simulations but kept the isoform abundance constant (Supplementary Figs. 3 and 4). See Supplementary Note 2 for a complete description of our simulation.

IsoCon’s precision increases with increased read depth, even when the average read depth per transcript is as high as 1562x (Supplementary Fig. 1). Such robustness is often hard to attain because increases in coverage beyond what is necessary for recall will only increase the number of errors in the data. The recall depends on the error rate influenced by the gene length. For *TSPY*, with equal abundance rates, the recall becomes perfect at 17x coverage, while for *DAZ*, the recall reaches >90% at only about 410x coverage (Supplementary Fig 3). We expect accuracy to also be a function of gene copy similarity, i.e. a gene family that is generated using low mutation rate, thereby producing fewer variants between gene copies, has the potential to negatively affect IsoCon’s ability to separate transcripts. Somewhat surprisingly, accuracy decreases only slightly in these cases, and read depth has a much more substantial effect on accuracy than mutation rate or gene length.

**Fig. 2:**
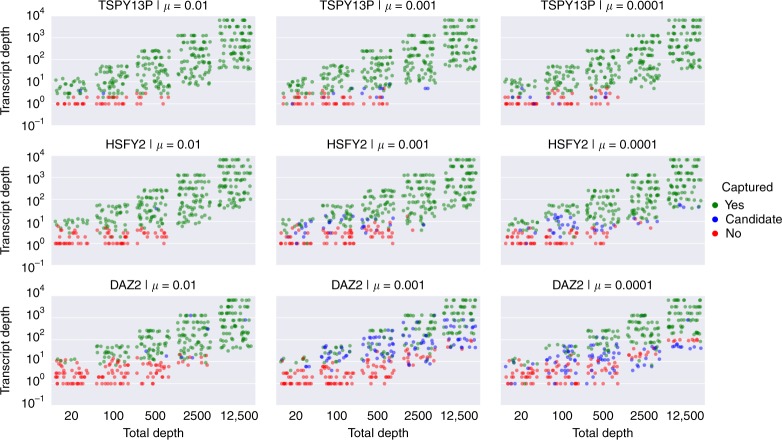
Power of IsoCon to capture transcripts. Each isoform from the experiments in Supplementary Figure 1 (including the 10 simulated replicates) corresponds to a marker in this plot which is marked according to whether it is captured and output as a final prediction (green), derived in the error correction step but filtered out in the statistical step (blue) or not produced at all in the correction phase (red) by IsoCon. This is a stripplot generated with the seaborn package^64^, which is a special kind of dotplot where the *x*-axis is categorical (total number of reads of the corresponding experiment) and points are spread out horizontally. The *y*-axis shows the number of reads that were sequenced from the isoform, on a log scale. Isoforms that have no reads are not shown

Our experiments clearly indicate that IsoCon’s recall is strongly dependent on read depth. We investigated this in more detail by taking every transcript simulated as part of the experiments in Supplementary Figure 1. We show (Fig. 2) whether or not IsoCon captured a transcript as a function of the sequencing depth (i.e. total number of reads) of its respective experiment and its own sequencing depth (i.e. the number of reads that were sequenced from the isoform). For *TSPY*, IsoCon captures most transcripts with depth >3, while this number is ∼10 for *HSFY*. The mutation rate plays only a minor role compared to the transcript depth as most candidates with lower read depth are lost in the error correction step (Fig. 2). We also observe that for *DAZ*, the minimum transcript depth required to capture an isoform increases as the total sequencing depth increases suggesting that relative transcript abundance is also a factor. This is likely due to the fact that the multi-alignment matrix becomes increasingly noisy (particularly for *DAZ*), as the number of sequences grows, negatively impacting both the error correction and the support calculation in the statistical test.

We also observe that IsoCon outperforms ICE in both precision and recall (Fig. 1, Supplementary Figs. 1, 3–5). ICE has poor precision which decreases with increased read depth. For example, at a read depth of 500 or higher, ICE’s precision is close to 0 in all our experiments. On the other hand, IsoCon’s precision is always >80% at read depths of 500 or higher. IsoCon also has higher recall in almost all cases for *HSFY* and *DAZ*. As for *TSPY*, the recall advantage fluctuates between the two algorithms but is fairly similar overall. Further investigation of IsoCon’s performance is detailed in Supplementary Note 3.

### Data from two human testes samples

We generated RT-PCR targeted transcript sequencing data for nine ampliconic gene families for two human male testes samples using the Iso-Seq protocol from PacBio (Supplementary Fig. 6 shows the number of passes per read) and, separately, using Illumina sequencing technology. We then used the ToFU pipeline^18^ to filter out any PacBio CCS reads that either were chimeric or did not span a transcript from end to end. We refer to the resulting set as the original (CCS) reads. For the purposes of comparison, we ran IsoCon and ICE^18^ on the CCS reads. We also evaluated the proovread^48^ tool, which uses Illumina reads to correct CCS reads (referred to as Illumina-corrected CCS reads). Supplementary Note 4 provides details on how these tools were run. We compared the results of the three approaches, as well as of the approach of just using the original CCS reads. Table 2 shows the number of reads generated and the number of transcripts called by each of these four different approaches.

**Table 2:** The number of reads and predicted transcripts. We show: (1) maximum RT-PCR product length per family (including primers), (2) the number of original PacBio CCS reads, with the number of distinct read sequences in parentheses, (3) the number of Illumina reads, (4) the number of distinct proovread Illumina-corrected CCS reads, (5) the number of ICE-predicted transcripts, and (6) the number of IsoCon-predicted transcripts. We use s1 and s2 to indicate sample 1 and sample 2, respectively

Family	Max length (nt)	Original PacBio CCS reads (s1)	Illumina reads (s1)	Illumina-corrected CCS (s1)	ICE (s1)	IsoCon (s1)	Original PacBio CCS reads (s2)	Illumina reads (s2)	Illumina-corrected CCS (s2)	ICE (s2)	IsoCon (s2)
*BPY*	321	36 (22)	6854	22^a^	1	2	37 (15)	9916	15^a^	1	1
*CDY_1* ^b^	1660	1110 (1090)	55,228	508	72	28	453 (439)	41,434	184	18	5
*CDY_2* ^c^	1623	442 (440)	75,862	322	19	11	1766 (1670)	74,080	630	28	28
*DAZ*	2235	495 (487)	49,500	208	14	34	530 (519)	39,318	291	16	34
*HSFY*	1163	933 (877)	14,832	350	26	25	205 (181)	26,408	59	5	2
*PRY*	421	177 (126)	40,904	121	8	8	25 (20)	6864	20	4	3
*RBMY*	1483	6615 (6365)	85,068	3698	105	162	6939 (6284)	65,284	2840	90	181
*TSPY*	916	2121 (1955)	27,428	903	32	133	1418 (1249)	8756	772	36	80
*VCY*	378	50 (23)	11,820	23	2	2	53 (47)	3328	47	1	7
*XKRY*	340	53 (28)	15,722	28^a^	2	1	55 (39)	2890	39^a^	1	3
Total	N/A	12,032 (11,413)	383,218	6183	281	406	11,481 (10,463)	278,278	4897	200	344

^a^proovread exited with an error that the sequences are too short and was not able to correct any reads

^b^*CDY* transcripts captured using the first primer pair (see Supplementary Fig. 11 for details)

^c^*CDY* transcripts captured using the second primer pair (see Supplementary Fig. 11 for details)

### Validation

To validate IsoCon and compare its accuracy against other methods we used (1) Illumina reads, (2) internal consistency between samples, and (3) agreement with a transcript reference database.

**Fig. 3:**
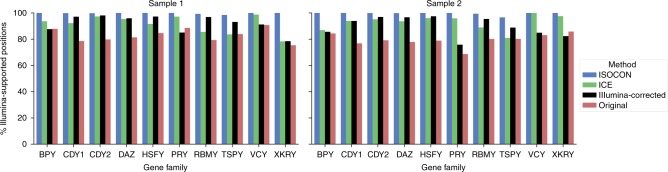
Illumina-support of predicted transcripts. Barplot shows percentages of positions in IsoCon/ICE/proovread, and original CCS flnc reads that are supported by at least two Illumina alignments with the same nucleotide

We validated the nucleotide-level precision of IsoCon, ICE, Illumina-corrected CCS reads, and original reads with Illumina data generated for the same two individuals. Throughout all positions in the predicted transcripts, we classified a position as supported if it had at least two Illumina reads aligning to it with the same nucleotide as the transcript. Since Illumina sequencing depth was orders of magnitude higher than that of PacBio (Table 2), we expect most correct positions to be supported. Note that the lack of Illumina support does not always indicate an error, since Illumina’s GC-bias will result in some regions being unsequenced. However, we expect that the number of transcript errors to be correlated with the number of unsupported positions. Fig. 3 shows the percentages of supported nucleotides for each approach. On average, 99% of IsoCon transcript positions are supported, but only 93% of ICE transcript positions are supported. Similarly, 96% of Illumina-corrected read positions and 79% of original read positions are supported. Furthermore, IsoCon has 70% of its transcripts fully supported (i.e. at every single position) by Illumina, compared to 2% for ICE, 15% for Illumina-corrected reads, and 20% for uncorrected reads.

While we expect some variability in the transcripts present in the two samples, we also expect a large fraction of them to be shared. IsoCon detected 121 transcripts that are present in both samples, corresponding to 32% of total transcripts being shared (averaged between two samples; Supplementary Figure 7). The Illumina-corrected CCS reads shared 11%, while both ICE and the original reads shared less than 2%. This likely indicates the higher precision of IsoCon relative to other methods.

**Fig. 4:**
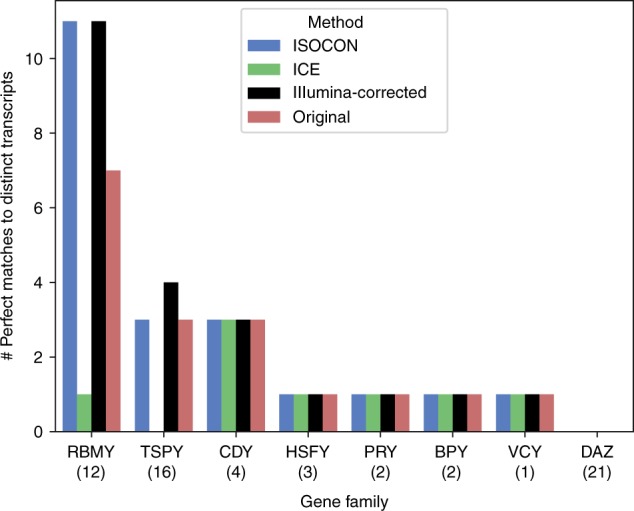
Ability of the methods to capture reference protein-coding transcripts in ENSEMBL database. The numbers in parentheses next to the gene family name in the *x*-axis indicate the number of unique transcripts in the database. No coding sequences are present for *XKRY*

IsoCon also did a better job at recovering known Ensembl transcripts. We downloaded annotated coding sequences of the nine Y chromosome ampliconic gene families from the Ensembl database^52^, containing 61 unique transcripts after removing redundancy (see Supplementary Note  5). We then identified database transcripts that were perfectly matched by the predicted transcripts. IsoCon had 21 matches to Ensembl, while ICE had only eight (included in those matched by IsoCon; Fig. 4). IsoCon also had more matches to the database than the original reads, despite reducing the number of sequences by a factor of >29 (Table 2). Illumina-corrected CCS reads in total had one more exact match than IsoCon, but had >14 times more predicted transcripts to IsoCon, suggesting low precision.

We also investigated the accuracy of IsoCon transcripts that had higher significance *p*-values and found that, while the accuracy is slightly decreased relative to transcripts with lower *p*-values, it still remains substantially higher than ICE or Illumina-corrected CCS reads (Supplementary Note 5).

### Isoform diversity

**Fig. 5:**
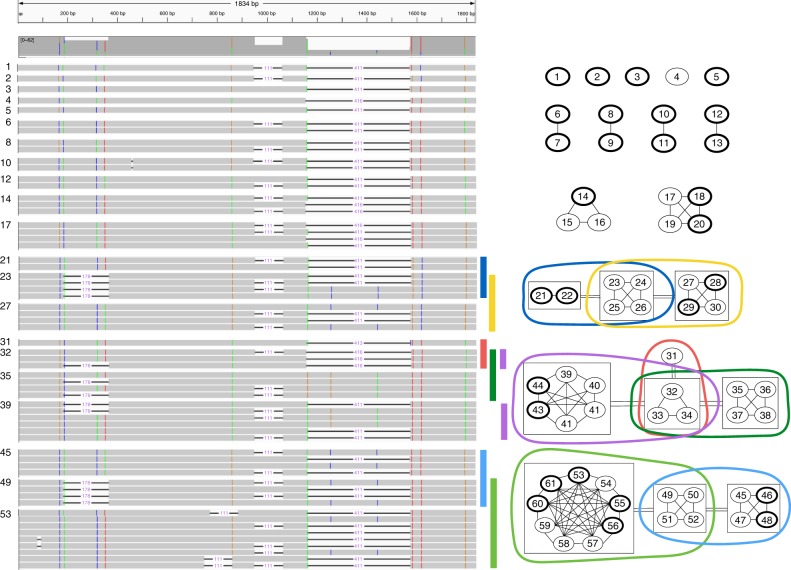
Illustration of the relationship between the 61 RBMY transcripts predicted by IsoCon and shared by both samples. Transcripts are indexed from 1 to 61. The left part of the figure uses IGV^65,66^ to visualize a multiple-alignment of the transcripts. Colored positions are positions with variability in the transcripts while grey regions depict conserved positions. Deletions are shown with a horizontal line, with a number indicating their length. The right part of the figure illustrates the relationship between the 61 transcripts as a graph. Vertices are transcripts (labelled with their indices). A vertex is boldfaced if it is predicted to be protein-coding. An edge between two transcripts means that they are potential isoforms from the same gene copy, i.e., they have only exon presence/absence differences. To simplify the visualization, some of the vertices are surrounded by boxes, and a double-edge between two boxes indicates that all pairs of transcripts, between the two boxes, are potential isoforms from the same gene copy. Each maximal clique (i.e. group of vertices) greater than four vertices is shown as a colored circle. The colors of the circles correspond to the rows in the multiple-alignment that are marked with a similarly-colored vertical bar. A maximal clique should be interpreted as all transcripts that potentially originate from the same gene copy

To study the transcripts IsoCon found, we first filtered out transcripts that were detected in only one sample. While we expect variability between the two individuals, such transcripts could also be false positives arising due to reverse transcriptase PCR (RT-PCR) errors^53^. These errors, if present, were introduced prior to library construction and would be located in both Iso-Seq and Illumina reads. They would lead to unique sequences that would mimic true transcripts in both Iso-Seq and Illumina data. Our downstream analysis only uses the 121 transcripts that were identically predicted by IsoCon in both samples. This removes any RT-PCR errors present in only one sample and reduces any false positive transcripts due to uncorrected sequencing errors. Table 3 shows the number of shared transcripts separated into gene families. We note, however, that the true number of transcripts in a sample might be higher due to sample-specific variants that we discarded. We further classified each transcript as protein-coding or non-coding depending on whether it is in-frame or out-of-frame with the human reference transcripts (see Supplementary Note 5 for details). We found that 72 out of the 121 transcripts are coding, and five of the nine families harbor a total of 49 non-coding transcripts (Table 3), the other four families have only coding transcripts. We also found that 94 out of the 121 transcripts were not previously known (Table 3), i.e. did not have a 100% match spanning the whole transcript when aligned to NCBI’s non-redundant nucleotide database (*nr*/*nt*). The multi-alignment for IsoCon’s *RBMY* transcripts—the family with the most predicted transcripts—is shown in Fig. 5.

**Table 3:** IsoCon-predicted transcripts of the nine ampliconic gene families. Columns show predictions shared between samples, separated by gene family and categorized as coding or non-coding. The calculated number of groups is shown, in comparison to known copy numbers from the reference genome and observed in human populations. Novel transcripts are those that do not have a perfect alignment to the NCBI transcript reference database, the numbers in parentheses indicate additional transcripts that have a perfect alignment only to ESTs, synthetic constructs, or in silico-predicted transcripts

Gene family	Number of coding members annotated in the ref. ^67^	Range of copy numbers observed in human populations^68,69^	IsoCon transcripts shared between samples, coding	IsoCon transcripts shared between samples, non-coding	Total number of inferred groups	Number of inferred coding groups	Novel transcripts	Predicted novel splice variants, coding	Predicted novel splice variants, non-coding
*BPY*	3	2–3	1	0	1	1	0 (0)	–	–
*CDY*	4	2–4	3	2	2	2	1(1)	–	–
*DAZ*	4	2–5	5	1	3	3	5(0)	4	1
*HSFY*	2	2	0	0	–	–	–	–	–
*PRY*	2	2–3	1	2	1	1	2(0)	–	2
*RBMY*	6	6–18	26	35	18	14	49(3)	2	10
*TSPY*	35	12–38	34	9	20	14	37(3)	4	2
*VCY*	2	2–3	1	0	1	1	0(0)	–	–
*XKRY*	2	2–3	1^a^	0	1	1	0(0)	–	–
Total	32	32–79	72	49	51	38	94	10	15

^a^The *XKRY* transcript shared by two samples for *XKRY-*based primers had a better alignment to *XKR3* than to *XKRY* and thus might not be Y-specific, but we did find a Y-specific transcript unique to sample 2

### IsoCon is sensitive to small and low-abundance variation

IsoCon was able to detect several transcripts even in the presence of an isoform with a much higher abundance that differed by as little as 1–3 bp. For example, one *RBMY* transcript that IsoCon recovered in sample 2 was supported by only five reads and differed by only one nucleotide from a transcript that was supported by 863 reads. A second example is another IsoCon *RBMY* transcript that was supported by only five reads in sample 2 and differed by only one nucleotide from a transcript that was supported by 306 reads. Both of these lower-abundance transcripts were derived in both samples (the support for these transcripts in sample 1 was 10 and 9 reads, respectively), had perfect Illumina support, and were protein-coding. Neither of these were detected by ICE or present without errors in the original reads; however, both of them were also derived in the Illumina-corrected CCS reads. Fig. 5 shows these two lower-abundance transcripts, indexed 3 and 1, respectively.

### Separating transcripts into gene copies

A gene family consists of gene copies, each of which can generate several isoforms because of alternative splicing. In such cases, the transcripts will align to each other with large insertions/deletions (due to missing exons) but without substitutions. We determine the minimal number of groups (i.e. clusters) that are required so that each transcript can be assigned to at least one group and every pair of transcripts in the same group differs only by large insertions/deletions (see Supplementary Note 5 for details). We refer to this as the number of groups, which is our best estimate of the number of copies, i.e. the size of each gene family. Note that allele-specific transcripts are non-existent for Y ampliconic gene families because of the haploid nature of the Y chromosome. We also determine the number of coding groups, which is the number of groups computed from the coding transcripts only. A group corresponds to the notion of a maximal clique from graph theory^54^, and since the number of transcripts is relatively small, the number of groups can be computed using a brute-force algorithm (Supplementary Note 5).

The number of groups for the nine different families are shown in Table 3, and Fig. 5 illustrates the idea of groups using the *RBMY* family as an example. An important distinction between a group and a gene copy is that a transcript can belong to multiple groups. This happens if an exon that contains a variant separating two gene copies is skipped over during the splicing process. In such a case, we cannot determine which copy the transcript originates from, and our approach places it in both groups. The size of each group is therefore an upper bound estimate of the number of isoforms originating from each gene copy.

We note that the true number of copies in a gene family might be higher or lower than the number of groups determined by IsoCon, for several reasons. First, it is impossible to separate transcripts originating from copies with identical exonic sequences. As a result, we might underestimate the true number of copies. Second, copy number might differ between the two males analyzed^55^. Because we are excluding transcripts unique to each male, we might underestimate the true copy number. Also, the copy number for a gene family might be the same between the two men, but some of the copies might have different sequences. Third, there may be copies that biologically differ only by the presence/absence of exons or by other large indels—if there are no substitutions, the resulting transcripts would be grouped together and treated as originating from the same gene copy by our approach. Since most human Y chromosome ampliconic genes were formed by whole-region duplications^56^, this situation should not be common. Nevertheless, if present, it would underestimate the number of copies. Fourth, RNA editing may generate transcripts that have substitutions but originate from the same copy, leading to overestimating the true copy number. Fifth, our approach to group transcripts relies on accuracy of transcript-to-transcript alignments, which can sometimes be inaccurate in the presence of repeats. Sixth, our approach sometimes places a transcript into more than one group (as described above) and can hence inflate the number of isoforms originating from each gene copy. With these caveats in mind, we nevertheless expect the number of groups to be a useful proxy for the size of the gene family.

We compared our number of coding groups against the number of copies annotated on the Y chromosome in the human reference genome (GRCh38/ hg38) and observed in previous studies of DNA variation in human populations (Table 3). For one of the gene families (*HSFY*), we did not find any transcripts shared between individuals. For four gene families (*CDY*, *DAZ*, *RBMY*, and *TSPY*), the number of coding groups falls within the previously observed range based on DNA analysis in human populations (Table 3). For the remaining four gene families (*BPY*, *PRY*, *VCY*, and *XKRY*), the number of coding groups is less than the copy number reported by prior studies. Three of these families—*BPY, VCY* and *XKRY—*had only one coding transcript shared between the two samples. Thus, overall, the number of coding groups is a conservative estimate of the number of gene copies per ampliconic gene family.

### Novel splice variants

For IsoCon’s 121 predictions that were shared between samples, 38 transcripts indicated novel variations in intron–exon start coordinates (Supplementary Table 1, details of analysis in Supplementary Note 4). This includes 21 “novel-in-catalog” transcripts (defined in ref. ^57^ as “containing new combinations of previously known splice junctions or novel splice junctions formed from already annotated donors and acceptors”) and 17 transcripts with containing at least one splice junction that did not align to any known splice junction (corresponding to either a novel splice junction or to a gene copy not represented on hg19). All 21 novel-in-catalog transcripts had strong Illumina support (at least four high-quality full length Illumina alignments) across the splice junctions and aligned to hg19 without substitutions or indels near the splice junctions (giving us confidence in the alignments near splice junctions). Out of the other 17 transcripts, 15 had strong Illumina support across the potentially novel splice junctions.

Some transcripts differed from each only in small intra-exon mutations but not in the splice pattern. Thus, the 38 transcripts correspond to 25 unique novel splice patterns, of which 13 represent novel-in-catalog splice patterns. Out of the 25, 10 are coding and 15 are non-coding (Table 3).

### Data from the *FMR1* gene

Targeted Iso-Seq was performed in ref. ^49^ on the *FMR1* gene in three carriers (premutation carriers for Fragile X-associated Tremor/Ataxia Syndrome), each sequenced with three SMRT cells, and three controls, each sequenced with one SMRT cell. The ToFU pipeline (using ICE for clustering and error correction) was used in conjunction with alignments to a reference genome to derive 49 total isoforms which were present in at least one of the six samples. Three additional isoforms were predicted in an earlier study^58^, but concluded missing in ref. ^49^. We use these 52 isoforms to benchmark IsoCon’s performance. Note that Tseng and colleagues were primarily interested in the splicing structure and did not study point mutations or indels. Therefore, we did not analyze these aspects of IsoCon results. For analysis details, see Supplementary Note 6.

When looking at the presence/absence of the 49 isoforms in each of the samples, IsoCon detected an average of 24 isoforms per sample (Supplementary Table 3), while Tseng and colleagues detected only 20 (Supplementary Table 9 in^49^). IsoCon also detected two out of the three predicted isoforms from^58^, which were not found in ref. ^49^. Out of the 24 isoforms predicted to exist for this gene by Pretto and colleagues, this brings the total number confirmed to 23.

One of the interesting findings of ref. ^49^ is that out of the 46 isoforms found in the carriers, 30 were not found in any of the control samples. Tseng and colleagues speculated about the potential role of this in detrimental protein activity. However, IsoCon was able to detect 5 out of these 30 in the controls. While the majority of isoforms were still specific to the carriers, our findings suggest that the difference may be smaller than found in ref. ^49^.

Many of the isoform occurrences that were detected by IsoCon but not by Tseng and colleagues were present at low coverages (Supplementary Table 3). For example, the extra five transcripts detected by IsoCon in the controls had a low number (3–9) of reads supporting them. We generally observed that IsoCon had a better relative performance in detecting isoforms for the control samples, which were sequenced at one-third the depth of the cases. Specifically, for the three controls, IsoCon detected an average of 17 isoforms per sample, while Tseng and colleagues detected only 12 (compared to the carriers, where the average number of isoforms detected was 31 and 27, respectively). Our findings suggest that IsoCon is more sensitive at lower coverages than ICE.

Additionally, IsoCon’s nucleotide-level resolution is useful to detect novel splice sites. For example, we found a novel splice variant belonging to isoforms in group C (as classified in ref. ^49^). IsoCon detected the splice variant in all of the three premutation carriers and in one of the controls. The transcript harboring the novel splice junction differs from previously derived isoform7 in group C by having a splice site starting five base pairs downstream to the start of exon 17, see Supplementary Figure 8a. While the *FMR1* dataset has no Illumina reads to validate the splice junction, there are several factors suggesting it is a bona fide isoform^23^: the new splice site is canonical (i.e., GT-AG intron flanks), the transcript has no indels or substitutions in the alignment to hg19, and it has high CCS read support (>105 in each of the three premutation carriers). Similarly, we observed a novel splice junction resulting from a deletion of the last nucleotide in exon 11 which were present in four isoforms in groups C and D and supported by thousands of CCS reads (Supplementary Fig 8b). Such predictions generated by IsoCon can provide candidates for downstream studies.

## Discussion

We have used both simulated and experimental data to demonstrate the performance of IsoCon in deriving transcripts from high-identity multi-gene families. This problem could algorithmically be seen as a generalization of deriving the allele-specific variants of a gene. There is however no functionality implemented in IsoCon that would distinguish allele-specific variants from distinct gene copies. We do, however, believe that IsoCon would be suitable to derive allele-specific transcripts. In such a scenario, further downstream analysis would be needed if one desired to distinguish different alleles from distinct copies.

The experimental methods we use here have some potential limitations. First, very low-abundance transcripts might not be captured by Iso-Seq, since deep PacBio sequencing can be cost-prohibitive. This limitation can potentially be overcome via augmenting Iso-Seq data with Illumina RNA-seq data and modifying IsoCon to incorporate such data. Second, our approach of only sequencing the transcriptome does not provide a definitive answer to the size of each gene family and does not allow us to conclusively assign each transcript to a gene copy. Similarly, it is difficult to distinguish novel gene copies from splice variants or RNA-editing. To alleviate this, one can amplify and sequence exons from the same individual, and compare RNA-derived and DNA-derived sequences. Third, though we see very good Illumina support for IsoCon’s transcripts, we cannot exclude the possibility of other error sources, such as RT-PCR errors. To allay this, two replicate cDNA libraries can be prepared from the same sample, though further validations will still be needed. Finally, a PCR-based target enrichment approach, which we utilized here, might not have captured transcripts of gene copies with mutations at PCR primer sites. An alternative to this approach is to use a probe-based capture technique^59^ which does not depend on PCR primers.

These limitations notwithstanding, IsoCon has allowed us to detect an unprecedented number of isoforms, many of them novel, as well as to derive better estimates on the number of gene copies in Y ampliconic gene families. IsoCon can also be useful for deciphering isoforms from genes with significant alternate splicing, such as *FMR1*. IsoCon is also sensitive to minor shifts in the splice junctions. For example, three *RBMY*-transcripts (19, 31, and 33 in Fig. 5) were splice variants that differ by only a 3–5nt splice difference to closest matching transcript; IsoCon found similar variants in the *FMR1* data (Supplementary Fig. 8). While such predictions still need to be validated, they enable further functional analysis and are expected to open novel avenues of Y ampliconic genes research.

## Methods

### Overview of IsoCon

The input to the IsoCon algorithm is a collection of PacBio CCS reads with at least one full pass through the transcript, and their base quality predictions. IsoCon assumes the reads have been pre-processed with the Iso-Seq bioinformatic pipeline to remove primers, barcodes, and reads that are chimeras or do not span the whole transcript. The pre-processing step separates the reads according to the primer pairs used to amplify individual gene families, and IsoCon is run separately on each gene family. The output of IsoCon is a set of transcripts which are the result of error-correcting the reads and reporting each distinct read.

IsoCon consists of two main steps: (i) an iterative clustering algorithm to error-correct the reads and identify candidate transcripts, and (ii) iterative removal of statistically non-significant candidates.

The clustering/correction step partitions the reads into clusters, where reads that are similar group together into one cluster. A multiple alignment and a consensus sequence is computed for each cluster. The reads in each cluster are then partially error-corrected to the cluster’s consensus sequence; to avoid removing true variants, only half of the potentially erroneous columns are corrected. Then, the process iterates—the modified reads are repartitioned into potentially different clusters and corrected again. This process is repeated until no more differences are found within any cluster, and the distinct sequences remaining are referred to as candidate transcripts (or simply candidates).

The clustering/correction step is designed to be sensitive and is therefore followed by the second step, which removes candidate transcripts that are not sufficiently supported by the original (uncorrected) reads. Initially, the original reads are assigned to one of their closest matching candidates. Then, evaluating all pairs of close candidates, for every pair we check whether there is sufficient evidence that their assigned reads did not in fact originate from the same transcript. To do this, we take two candidates and the set of variant positions (i.e., positions where the two candidates differ) and formulate a hypothesis test to infer how likely it is that the reads supporting these variants are due to sequencing errors. Since a candidate can be involved in many pairwise tests, it is assigned the least significant *p*-value from all pairwise tests performed. After all pairs of candidates have been tested, a fraction of non-significant candidates will be removed. The second step of IsoCon is then iterated—the original reads are assigned to the best match out of the remaining candidates, which are then statistically tested. This continues until all remaining candidates are significant. The remaining candidates are then output as the predicted transcripts.

### Clustering and error correction step

First, we need to define the concept of closest neighbors and the nearest neighbor graph. Let dist(*x*,*y*) denote the edit distance between two strings *x* and *y*. Let *S* be a multi-set of strings. Given a string *x*, we say that a *y*∈*S* is a closest neighbor of *x* in *S* if ${\mathrm {dist}}\left( {x,y} \right) = \mathop {{{\mathrm {min}}}}\limits_{z \in S} {\mathrm {dist}}\left( {x,z} \right)$. That is, *y* has the smallest distance to *x* in *S*. The nearest-neighbor graph of *S* is a directed graph where the vertices are the strings of *S*, and there is an edge from *x* to *y* if and only if *y* is a closest neighbor of *x* but is not *x*.

There are two phases to the clustering/correction step—the partitioning phase and the correction phase—and we iterate between the phases. In the partitioning phase, we first partition the reads into clusters, with each cluster having exactly one read denoted as the center. The idea is that each partition contains a putative set of reads that came from the same transcript and the center is the read whose sequence is most similar to that of the transcript. To partition, we first build a graph *G* which, initially, is identical to the nearest-neighbor graph built from the reads. Next, we identify a read *x* in *G* with the highest number of vertices that can reach *x*. We create a new cluster with *x* as the center and containing all reads in *G* that have a path to *x*, including *x* itself. Next, we remove the elements of the new cluster, along with their incident edges, from *G*. Then, we iterate on the newly modified *G*: identifying the vertex with the highest number of vertices that can reach it and creating a cluster centered around it. The full pseudo-code is given in the PartitionStrings algorithm in Supplementary Fig. 9.

The resulting partition has the property that each string has one of its closest neighbors (not including itself) in its cluster. This closest neighbor may be the center but does not have to be. Thus, a cluster may contain many strings which are closest neighbors of others but only one of them is denoted as the center.

The correction phase works independently with each cluster of reads and its corresponding center. We first create pairwise alignments from each read to the center using parasail^60^. We then create a multi-alignment matrix *A* from the pairwise alignments (for details see Supplementary Note 1). Each entry in *A* is either a nucleotide or the gap character, and each row corresponds to a read. We obtain the consensus of *A* by taking the most frequent character in each column. Every cell in *A* can then be characterized as one of four states with respect to the consensus: a substitution, insertion, deletion, or match. Given a column *j* and a state *t*, we define $n_j^t$ as the number of positions in column *j* that have state *t*. Similarly, let *n*^*t*^ denote the total number of cells with state *t* in *A*. The support for a state *t* in column *j* is defined as $n_j^t/n^t$, and the support of a cell in *A* is the support for that cell’s state in that cell’s column. The support is state specific to be more sensitive to distinct error types. For example, as deletions and insertions are frequent, more coverage is needed for these variants not to be corrected, compared to a substitution. Next, in each read, we identify the variant positions (i.e. whose state is substitution, insertion, or deletion) and select half of these positions that have the lowest support. Then, for each of these positions, we correct it to the most frequent character in the column; but, if the most frequent character is not unique, then no correction is made.

IsoCon’s clustering/correction step combines the partitioning and correction phases in the following way. Initially, we partition the set of reads and correct each cluster. A cluster is said to have converged if all its strings are identical. As long as at least one cluster is not converged, we repeat the partitioning and correction phases. To ensure that eventually all clusters converge, we heuristically undo the correction of a string if, after correction, it has a higher edit distance to the center than it had to its center in the previous iteration, if the string alternates between partitions in a cyclic fashion, or the same set of strings repeatedly get assigned to the same partition where they differ only at positions where the most frequent character is not defined. Finally, after all the partitions have converged, we designate their centers as candidate transcripts and pass to the candidate filtering step of IsoCon. The full pseudo-code for this step is given in the ClusterCorrect routine in Supplementary Figure 9.

### Candidate filtering step

IsoCon’s second step takes as input a collection of reads *X* and a set of candidate transcripts $C = \{ c_1, \ldots ,c_l\}$. The first step is to assign reads to candidates, such that one read is assigned to exactly one of its closest neighbor candidates in *C*. Because a read may have several closest neighbor candidates in *C*, there are many possible assignments. For our purposes, we use the following iterative greedy algorithm. For each read *x*∈*X*, we identify its closest neighbor candidates in *C*. Next, we select a candidate *c*∈*C* that is a closest neighbor of the most reads in *X*. We assign all these reads to *c*, and remove *c* from *C* and all the assigned reads from *X*. We then repeat the process, using the reduced *X* and *C*, until all reads have been assigned.

Now, we have an assignment of reads to the candidates. We denote by *X*_*i*_ the reads that are assigned to candidate *c*_*i*_. We check for evidence to support that *c*_*i*_ is a true candidate as follows. We consider the candidates who are the closest neighbors of *c*_*i*_ in $C - \{ c_i\}$. Next, for each closest neighbor candidate *c*_*j*_, we form the null-hypothesis that the reads in *X*_*i*_ and in *X*_*j*_ originated from *c*_*j*_, i.e. *c*_*i*_ is not a true candidate. The significance value calculation under this null-hypothesis is given in the next section. We compute *p*_*i*_, the least significant value, amongst all *c*_*j*_. We limit our comparisons of *c*_*i*_ to only its closest neighbor candidates because it keeps our algorithm efficient and it is unlikely that comparison against other more dissimilar candidates would increase *p*_*i*_.

Then, we identify the candidates with *p*_*i*_ greater than a significance threshold *α*. This *α* is a parameter to our algorithm, set by default to 0.01. These candidates are then removed from the candidate set *C*. Given a parameter *τ*, if there are more than *τ* candidates with significance value over *α*, we only remove the top *τ* candidates with the highest values. The candidate filtering step of the algorithm then iterates: we again assign reads to candidates and identify candidates with insufficient support according to our hypothesis test. The algorithm stops when there are no longer any candidates with *p*_*i*_ above *α*. The pseudo-code for this algorithm, together with all of IsoCon, is given in Supplementary Figure 9.

### Statistical test

We are given two candidate transcripts *c* and *d* and sets of reads *X*_*c*_ and *X*_*d*_ that have been assigned to them. We use $x_i \in X_c \cup X_d$ to denote each read and let *n* be the number of reads in $X_c \cup X_d$. We calculate pairwise alignments from $X_c \cup X_d \cup \{ c\}$ to *d*. Next, we construct a multi-alignment matrix *A* from these pairwise alignments in the same way as for the correction step (for details see Supplementary Note 1). Each entry in *A* corresponds to either a nucleotide or the gap character. Let *V* be the index of the columns of *A* where *c* and *d* do not agree. We refer to these positions as variant positions.

Let *A*_*i,j*_ denote the character in column *j* of row *i* of *A*. Rows $1 \le i \le n$ correspond to reads *x*_*i*_, while row *n* + 1 corresponds to *c*. For $1 \le i \le n$, we define a binary variable *S*_*i*_ that is equal to 1 if and only if *A*_*i,j*_ = *A*_*n*+1,*j*_ for all *j*∈*V*. That is, *S*_*i*_ is 1 if and only if read *x*_*i*_ supports all the variants *V*, i.e. has the same characters as *c* at positions *V* in *A*. We make the following assumptions:

1. *d*, acting as the reference sequence in this test, is error-free.
2. A nucleotide in a read at a position that is not in *V* and differs from the corresponding nucleotide in *d* is due to a sequencing error. In other words, at position where *c* and *d* agree, then they cannot be both wrong.
3. The probabilities of an error at two different positions in a read are independent.
4. *S*_*i*_ and $S_{i\prime }$ are independent random variables for all $i \ne i\prime$.

Our null-hypothesis is that the variant positions in *A* are due to sequencing errors in *X*. To derive the distribution of *S*_*i*_ under the null-hypothesis, we first need a probability, denoted by *p*_*ij*_, that position *j* in read *i* is due to an error. This can largely be obtained from the Phred quality scores in the reads (see Supplementary Note 1 for details). Under assumption 3 we have that *S*_*i*_ follows a Bernoulli distribution with mean $p_i = \mathop {\prod}\limits_{j \in V} {p_{ij}}$.

A relevant test statistic under the null-hypothesis is a quantity that models the strength (or significance) of support for variants *V*. We would like to only count reads that fully support all the variants, i.e. reads *x*_*i*_ with *s*_*i*_ = 1(*s*_*i*_ denotes the observed value of *S*_*i*_). These reads may have errors in non-variant locations, but, at the variant locations, they must agree with *c*. For each such read, we would like to weigh its contribution by the inverse of the probability that all the characters at the variant locations are due to sequencing errors. Intuitively, a read with a high base-quality should count as more evidence than a read with a low base-quality. Taking these considerations together, we define our test statistic as

$$\hskip 70ptT = \mathop{\prod}\limits_{i=1}^{n} \frac{1}{{\left( {P\left( {S}_{i} = {1} \right)} \right)^{S}_{i}}} = {}^{\left(under\ the\ null\right)}\mathop{\prod}\limits_{i=1}^{n} {\frac{1}{{p}_{i}^{s_{i}}}}$$

Notice that *p*_*i*_ decreases with the amount of variants in *V* and with higher base quality scores; therefore, $T$ is designed to leverage linked variants across the transcript, in the sense that less reads are required to support a transcript when the transcript has more variants. Moreover, $p_i$ decreases for reads with higher CCS base quality at variant positions, meaning less reads are needed to support a transcript, if they have higher quality. We observed that base quality values in the CCS was highly variable and depends on (i) the number of number of passes in a CCS read, (ii) the mono-nucleotide length, and (iii) the sequenced base, with C and G having lower qualities associated with them (Supplementary Fig. 10)

We let $t$ be the observed value of this statistic and we refer to it as the weighted support. Given $t$, we calculate a significance value as $P(T \ge t)$. We use a one-sided test as we are only interested in significance values of equal or higher weighted support for $V$. We are not aware of a closed form distribution of $T$ under the null-hypothesis, and a brute-force approach to calculating $P(T \ge t)$would be infeasible. However, we can make use of the following Theorem from ref. ^61^, which gives a closed formula upper-bound on the distribution of a sum of Bernoulli random variables:

Theorem: *Let* $a_1, \ldots ,a_n$ *be reals in* $(0,1]$ *and* $Z_1, \ldots ,Z_n$ *be Bernoulli random trials*. *Let* $Z = \mathop {\sum }\limits_{i = 1}^n a_iZ_i$ *and* $\delta > 0$ *and* $\mu = E(Z) \ge 0$, *then* $P(Z > (1 + \delta )\mu ) < \left( {\frac{{e^\delta }}{{(1 + \delta )^{1 + \delta }}}} \right)^\mu$.

In order to apply the Theorem to *T*, we must first make a log transformation to convert the product into a sum, and then normalize $T$ so that coefficients lie in $(0,1]$ as needed.

$$T\prime = \frac{{\log\, T}}{{\mathop {{{\mathrm {max}}}}\limits_k \left( { - \log\, p_k} \right)}} = \mathop {\sum}\limits_{i = 1}^n {\frac{{ - S_i\log\, p_i}}{{\mathop {{{\mathrm {max}}}}\limits_k \left( { - \log\, p_k} \right)}}}$$

The expected value is

$$E\left[ {T\prime } \right] = - \mathop {\sum}\limits_{i = 1}^n {\frac{{p_i\log\, p_i}}{{\mathop {{{\mathrm {max}}}}\limits_k \left( { - \log\, p_k} \right)}}}$$

Note that under this transformation, $P\left( {T\prime \ge t\prime } \right) = P\left( {T \ge t} \right)$, as the logarithm function is strictly monotone and the normalization using the maximum is constant. Let $\mu = E\left( {T\prime } \right)$ and $\delta = t\prime /\mu$. We can then apply the Theorem to obtain the bound

$$P\left( {T \ge t} \right) = P\left( {T\prime \ge t\prime } \right) < \left( {\frac{{e^\delta }}{{\left( {1 + \delta } \right)^{1 + \delta }}}} \right)^\mu $$

We use this upper bound as the significance value. Note that the Theorem only applies for $\delta > 0$. If $t\prime \le \mu$, then this is not the case. However, it implies that the observed weighted support is below the expected support, under the null-hypothesis. Such values are clearly insignificant, and our software defaults to a value of 0.5. Candidate transcripts that have more than a threshold of variant positions (default of 10) relative to all other candidate transcripts are not statistically evaluated because their *p*-value would be nearly 0.

### Relation to ICE

IsoCon, similarly to ICE, uses an iterative cluster and consensus approach, but the two algorithms have fundamental differences. After clustering, IsoCon derives a weighted consensus based on the error profile within a partition, and uses it as information to error correct the reads; ICE, on the other hand, derives a cluster consensus using the stand alone consensus caller DAGCON^62^, to be used in the next iteration without error correction of the reads. IsoCon and ICE also differ in the graphs they use to model the relationship between sequences and in the algorithm to partition the graph into clusters. IsoCon deterministically creates clusters modeled as a path-traversal problem, while ICE models a cluster as a maximal clique and uses a non-deterministic approximative maximal clique algorithm. Perhaps most importantly, IsoCon as opposed to ICE, includes a statistical framework that allows it to distinguish errors from true variants with higher precision.

### Experimental methods

Poly(A) RNA was isolated from testis RNA of two Caucasian men (IDs: CR560016, age 59, sample 1; CR561118, age 79, sample 2; Origene) using Poly(A) Purist MAG kit (Thermo Fisher Scientific). 50 ng of poly(A) RNA per each sample, along with 1 μg of control liver total RNA (used for control), were used to generate double-stranded DNA using SMARTer PCR cDNA Synthesis Kit (Clontech). PCR cycle optimization of cDNA amplification reaction using the Clontech primer was performed and 12 cycles were determined to be optimal for the large-scale PCR amplification. For each of nine ampliconic gene families, we designed a pair of RT-PCR primers with one primer located in the first, and the other primer located in the last, coding exon (Supplementary Table 4). For one of these gene families (*CDY*), an additional primer pair was designed to capture transcripts originating from all gene copies (Supplementary Fig. 11). One of the two unique PacBio barcodes was added to the primers in order to distinguish RT-PCR products between the two men. Next, RT-PCR products from these two individuals were separated into two equimolar pools according to the expected transcript sizes (<1 kb and 1–2 kb; Supplementary Table 4) and purified using AMPure XP beads (Beckman Coulter, Inc., USA). Each of the two RT-PCR pools was then used to construct a separate PacBio Iso-Seq library that was sequenced on RSII (P6-C4 chemistry) using one SMRT cell per library. Therefore, a total of two SMRT cells were sequenced.

Additionally, we sequenced the same RT-PCR products with Illumina technology. We constructed separate Nextera XT library (with a unique pair of indices) for each primer pair-sample combination. A total of nine gene families were analyzed with 10 primer pairs (as mentioned above, one gene family, *CDY*, was analyzed with two primer pairs). Therefore, 10 primer pairs × 2 individuals = 20 libraries were constructed. These libraries were normalized, pooled in equimolar ratio, and sequenced on a MiSeq instrument using one MiSeq Reagent Nano Kit, v2 (250 × 250 paired-end sequencing).

An extended version of the experimental protocol is available online at 10.1038/protex.2018.109.

### Code availability

IsoCon is open-source and freely available at https://github.com/ksahlin/IsoCon. The results of IsoCon in this paper were obtained with commit 79589f3 on GitHub. Detailed information of software parameters are given in Supplementary Note 4. Scripts for all analyses are available at https://github.com/ksahlin/IsoCon_Eval. This repository also include a snakemake^63^ workflow that reproduces intermediate and final data for the ampliconic gene family analysis.

## Electronic supplementary material

Supplementary Information

Description of Additional Supplementary Files

Supplementary Data 1

Supplementary Data 2

## Data Availability

All the long-read PacBio and short-read Illumina sequence data from two human males generated for this study have been deposited in NCBI Sequence Read Archive (SRA) under the BioProject accession number PRJNA476309. (https://www.ncbi.nlm.nih.gov/sra/SRP150854)
