# Supplementary material for: Deciphering highly similar multigene family transcripts from Iso-Seq data with IsoCon
Source: Nat Commun. 2018 Nov 2;9:4601. doi: 10.1038/s41467-018-06910-x (PMC6214943; doi:10.1038/s41467-018-06910-x)
Supplement: Supplementary file 1 — Supplementary Information [file 41467_2018_6910_MOESM1_ESM.pdf]

Supplementary Text

Deciphering highly similar multigene families with PacBio transcript data

Sahlin*, Tomaszkiewicz* et al.

Supplementary Note 1: IsoCon method details

In this note we describe some of the additional details about the method.

Combining pairwise alignments into a multi-alignment

In this section, we describe our heuristic algorithm to construct a multi-alignment matrix A from a set of pairwise alignments between a sequence c and sequences $x_i \in X$. Each entry in A corresponds to either a nucleotide or the gap character "-". Our approach is heuristic and may produce a multi-alignment that is not the optimal one according to a global scoring scheme. However, it is designed to work fast and be accurate on similar sequences. It also ensures that identical insertions relative to c are aligned in the same columns. This is crucial when using the alignment support of strings for both correction and statistical testing. But, we note that our strategy may not generalize well to alignments for more distant or repetitive sequences. In such cases, a more accurate method such as progressive multiple alignment may work better.

First, for every character of c, we construct the corresponding column of A. This is done in a straightforward manner, where row i contains the character of x_i that aligns to the character of c.

Second, we construct the columns of A that will contain a gap character in c, i.e. those columns where at least one sequence in X has an insertion with respect to c. Let $s_i(l)$ be the substring of $x_i$that is inserted between positions $l$and $l+1$ in c. Here, $s_i(0)$ and $s_i(|c|)$ will be any string inserted before the start or after the end of c. We let $s^{max}(l)$ be the longest insertion observed for position l, over all $x_i \in X$. If there is more than one longest insertion, we chose the smallest lexicographical string to avoid stochasticity.

For a given insertion site l, we pairwise align all $s_i(l)$ to $s_{max}(l)$, using the unit cost model but not allowing internal deletions in $s_{max}(l)$; that is, the only allowed deletions in $s_{max}(l)$ are prior to its first character or after its last character. To improve performance, we cache the computed pairwise alignments to avoid redundant alignment computations. From these pairwise alignments, we construct new columns in A, in between positions $l$and $l+1$ in c. After we do this for every insertion site, our multiple-alignment matrix A is complete.

Estimating the probability of a sequencing error

Recall that we are given a set of reads, a candidate to be tested c, and a reference candidate d. We align all the reads and c to d. We then build a multi-alignment matrix A of the reads and c. We denote the probability that position j in read i ($A_{i,j}$) is due to a sequencing error as p_{ij}. In this section, we compute p_{ij}, by multiplying the probability of an erroneous base call by the probability that the erroneous base call is exactly the character at position j. We derive these as a function of the Phred quality scores and the empirical distribution of the different types of errors (*error profile*) in the alignment matrix.

Each CCS read comes with a Phred base-quality score for each base. Let Q_{ij} be the Phred score of of read i at position j in the multi-alignment matrix. If $A_{i,j}$ is a gap character, then the quality of the nucleotide immediately to the right of the gap is used. By definition, a Phred quality score of Q_{ij} corresponds to a probability of an erroneous base call of $10^{Q_{ij}/10}$ [1]. The CCS caller in PacBio produces quality values in the range of $3 \leq Q_{ij} \leq 93$; however, we generally have found these can be overconfident estimates. Therefore, we remap them onto the range of $[3, T]$, with $T = 43$ (default), using the linear map $f(Q_{ij}) = (Q_{ij} - 3)\frac{T-3}{93-3} + 3$. With this adjustment, the probability of an erroneous base call is $q_{ij} = 10^{-f(Q_{ij})/10}$, which in practice means that no base call is allowed a Phred quality score larger than T.

Next, we need to compute the probability that the error results in character $A_{i,j}$, as opposed to some other character. Ideally, one would want the base caller to provide such information, but it is not currently provided. Instead, we use empirical estimates of the relative frequencies of different error types from the read's alignment. While not ideal, it does model the fact that the error profile across the reads is different for reads with different passes and in different homopolymer regions (Supplementary Fig. 2). According to the CCS base call uncertainty protocol [2], the only variant for which we can fully attribute the base-call uncertainty q_{ij} to a given error is when the variant is an indel in the first nucleotide of a homopolymer region. The uncertainty q_{ij} over that position reflects the uncertainty of the homopolymer length. We therefore attribute the whole uncertainty q_{ij} in those cases. For the other variants we are testing, let r_S, r_I, r_D be the relative frequencies of substitutions, insertions, and deletions in each read $x_i$in A. For a given variant at position j with a state of either substitution, insertion, or deletion in the candidate to be tested (c) with respect to the reference (d), we define p_{ij} as follows:

- $p_{ij} = q_{ij}$, if $j$is an insertion or deletion in homopolymer region ($\geq$ 2bases) in reference d

- $p_{ij} = q_{ij} * r_S/3$, if substitution
- $p_{ij} = q_{ij} * r_I/4$, if insertion (not in homopolymer region)
- $p_{ij} = q_{ij} * r_D$, if deletion (not in homopolymer region)

In some cases, Phred quality values are not available, e.g. in simulated data or if the CCS reads have been somehow post-processed prior to IsoCon. In this case we use the same formula as before but estimate q_{ij} empirically as the total number of mismatches (substitutions, deletions, insertions) in read i in its alignment to d, divided by the length of d. For a homopolymer of length h, since a deletion can happen in any of the h bases (similarly, insertion after any of h bases) we let the uncertainty be hq_{ij} in these regions. We have

- $p_{ij} = min(0.5, hq_{ij})$, if $j$is an insertion or deletion in homopolymer region (≥ 2 bases) in reference d
- $p_{ij} = q_{ij} * r_S/3$, if substitution
- $p_{ij} = q_{ij} * r_I/4$, if insertion (not in homopolymer region)
- $p_{ij} = q_{ij} * r_D$, if deletion (not in homopolymer region)

Implementation details

We avoid any non-determinism by always having explicit tie-breakers. For example, if the number of reachable vertices are the same for two vertices when partitioning the nearest neighbor graph in the PartitionStrings() routine (Supplementary Fig. 1), we take the one with the most number of neighbors. If still a tie, we chose the lexicographically smallest sequence.

We calculate edit distances using edlib [3] to find the closest neighbors, using the 'global' method. We then align with parasail, before error correction and statistical tests, with parameters gap_open=-2, gap_extend=0 match=2, and mismatch we set to either -1,-2 or -4 depending on how many percent the edit distance is to the total length of the alignment, <1%, <5% or above 5%. Note that higher edit distance have a higher mismatch score to favor indels, as they are more common for PacBio sequencing.

We do not assign reads to candidates in each iteration of the IsoCon algorithm (Supplementary Fig. 1, Algorithm 3, line 6) from scratch. Instead, we only realign reads assigned to a candidate that was filtered out.

We remove any edge in the nearest neighbor graph that was formed from an alignment with an internal gap of more than W nucleotides (parameter to IsoCon set to 20 by

default). Here, internal means that aligned sequence occur both before and after the gap. The intuition behind this is that a larger internal gap in a CCS read likely stems from an exon difference. The reason behind keeping edges that were formed despite gaps in ends is that sequences might be cut at different end positions in the Iso-Seq protocol. IsoCon also has a parameter to ignore smaller length differences on otherwise identically derived candidate transcripts (set by default to 15 base pairs). This is to account for the small variation in primer cutting-sites when they are removed. This parameter will, after the CCS read correction phase, treat all candidates that have identical sequences -- up to a start or end offset with less or equal to 15 base pairs -- as coming from the same transcript. In practice, the candidate with the highest CCS read support after the correction will be kept (most common primer cut-site), and the candidates with only an offset to this candidate are removed.

Supplementary Note 2: Generating simulated data

Generating gene families

To simulate a gene family with *m* member genes, we chose a reference gene as a starting point and download its exons from Ensembl[4]. We concatenate the exons in the order they appear on the genome. We use this artificial transcript as a start sequence, i.e., this is the root node in our evolutionary tree. We then branch the node into two children. We let one child be identical to its parent and in the other child we simulate mutations with mutation rate μ (a parameter). For each nucleotide, we simulate a substitution with probably $\mu/3$, a deletion with probability $\mu/3$, and an insertion with probability $\mu/3$. With probability $1 - \mu$, we do not alter the nucleotide. We then recursively continue the branching process for each child, in a breadth-first manner until we have *m* leaves. The sequences at the leaves are then taken as the gene family members. For a meaningful evaluation, if two members have identical sequences, we redo the simulation. The result is a set of *m* distinct gene family members with identical exon structures and where each exon may or may not be different between members.

Next, we can create *n* isoforms from a single gene by dropping exons as follows. Let *e* be the number of exons. One isoform is always taken to be the full set of exons. We can generate up to $e - 1$ additional isoforms by dropping one exon at a time. If $n > e$, then we can generate up to $e(e-1)/2$ more isoforms by dropping 2 exons at a time, and so on. We make sure that each isoform we generate is not identical to an isoform we generated for another gene in the family. This could arise if all the mutations that discriminate between the two genes lie in the dropped exons. In such cases, the isoform is skipped the next is one is generated. Our deterministic approach to dropping exons is motivated to make isoforms within and between copies as similar as possible, thereby giving the most challenge to an error-correction algorithm.

Finally, each isoform is given an abundance (i.e. the relative frequency of the isoform in the pool of transcripts). In the case of *equal abundance*, each transcript has the same abundance. In the case of *unequal abundance*, where we set the relative abundances of isoforms to be exponentially increasing as follows. Given a sequence of *n* isoforms $f_0, \ldots, f_{n-1}$, we first randomly permute them and then assign isoform f_i an unnormalized abundance of $2^{i \bmod 8}$. We then obtain the abundance frequency by dividing each unnormalized abundance by the sum of all the unnormalized abundances. For example, if there are 4 isoforms, the unnormalized abundances are 1, 2, 4, and 8, and the abundances are 1/15, 2/15, 4/15, and 8/15.

For each gene family and mutation rate in Figures 1 and Supplementary Figure 3, we simulated four gene family members and, for each member, we simulated 2, 4, 8, and 16 isoforms, respectively. For each gene family and mutation rate in Supplementary Figures 1 and 4, we simulate eight family members with one isoform each. For Figures 1 and 2, unequal abundances are used, while for Supplementary Figure 3 and 4, equal abundances are used. In each experiment, the number of simulated reads were also varied. We can obtain the isoform depth based on the relative abundance of isoforms. For example, in Figure 1, the isoform abundance ranges from 1/828 to 128/828. With 2,500 reads, the coverage ranges from 3.02x ($= 2500 * 1/828$) to 387x ($=2500 * 128/828$).

Generating PacBio CCS reads

Before implementing our own circular consensus (CCS) PacBio read simulator, we explored the possibility of using already existing PacBio read simulators such as PBSIM [5], LongISLND [6], and SiLiCO [7]. However none of the options were viable for our purpose of simulating full length non chimeric reads. SiLiCO does not simulate the circular consensus (CCS) protocol, which we use. PBSIM and LongISLND both assume genomic (i.e. not transcriptome) data. We tried both PBSIM and LongISLND with different parameters but were unable to get reads that would cover our full transcripts. This is because start and end positions in these tools are simulated to be within the reference sequence that are assumed to be a genome, while for our case the reference sequences are transcripts.

We therefore wrote our own PacBio CCS read simulator. The length of PacBio polymerase reads (i.e. the total length prior to the passes being collapsed by CCS) is simulated from a triangular distribution [8] to roughly mimic the shape of the polymerase read length distribution given in [9]. In the triangular distribution, we set smallest read length to 0 (start base of triangular distribution), the mode read length to 10,000 (top of triangle), and the largest read length to 45,000 (end of base in triangle). The polymerase lengths are generated from this distribution and the the number of passes of each read through a transcript is determined by this read length. We let the number of passes be $p = floor(L/n)$, where L is the polymerase length and n is the transcript length. Reads where $L<n$ are discarded, as we only use reads that span the whole transcript. The CCS read will then be simulated with nucleotide accuracy that corresponds to p passes using the reported PacBio CCS error rates [10]. For the case of just one pass, the simulated accuracy will be 13%, using the raw accuracy of the PacBio polymerase [9] and for more than 18 passes, we fix the error rate to 0.999[10]. When an erroneous nucleotide is simulated, the type of error is simulated according to published probabilities for PacBio reads without CCS [11] [12]: 68.75% for insertions, 25% for deletions and 6.25% for substitutions.

Computing recall and precision

To measure the accuracy of the results of an algorithm on the simulated data, we defined a true positive as a predicted sequence that is identical to a true isoform, a false positive as all other predicted sequences, and a false negative as a true isoform that is not present exactly in the predicted sequences. Matches were required to be exact, in order to capture nucleotide level accuracy. We then computed recall and precision based on these definitions.

Supplementary Note 3: Additional analysis of simulated data

We expect IsoCon's precision and recall to approach 1 at very high read depths. Supplementary Figure 1 shows accuracy at read depth of 12,500. In all except a few cases (described below), IsoCon successfully captures low abundant gene copies having edit distance 1 to other copies. This includes transcripts with abundance ratio 4:64 and 1:32 in TSPY, with μ=0.0001 and 0.001 respectively, and transcripts with abundance ratio 4:64 and 1:32 in HSFY and DAZ datasets with 0.0001 respectively (Supplementary Figure 1 and 2). There are two cases of false negatives. At mutation rate 0.0001, the lowest abundance transcript did not get captured in HSFY. This transcript has an edit distance of one to a copy to which it has an abundance ratio of 1:4 and an edit distance of two to a copy to which it has an abundance ratio 1:64 (Supplementary Figure 2). This presents a difficult case for the algorithm due to the presence of similar transcripts which are drastically more abundant. For DAZ, the transcript with the second lowest abundance is not captured in 9 out of the 10 replicated experiments, and it has an edit distance of one to a copy to which it has an abundance ratio 1:2 (Supplementary Figure 2). Further investigation showed that this transcript was not a closest neighbor of any node in IsoCon's error correction step, indicating room for future improvement.

We also evaluate IsoCon's and ICE's accuracy and recall on datasets with only one gene copy but several isoforms. We investigated this to confirm that there were no bias in separating transcripts on exon level. We investigated to separate 8 different isoforms for a copy where isoforms were samples with equal and unequal abundance. Recall and precision is shown in Supplementary Figure 5. As expected, since isoforms have different exon structures they are more dissimilar than gene copies differing in only mutations, and therefore, should be easier to capture compared to highly similar gene copies as in previous experiment. Supplementary Figure 5 confirms this for IsoCon. For ICE this is also true in general except for the DAZ datasets with 125000, panel A and B. ICE's recall drops significantly and we are unable to determine the reason for this.

Somewhat counterintuitively, IsoCon's recall is higher for lower mutation rates for the lowest read depth datasets for HSFY and DAZ (Supplementary Figure 5A, 20 reads for HSFY, 20 and 100 reads for DAZ). This is because with low read depth, IsoCon will cluster the highly similar copies into the same partition and, therefore, get increased coverage to recover one of the copies. For the more divergent cases, this cluster merging—giving increased coverage—will not happen and both the copies are instead lost. P-values of all IsoCon's predictions for the simulated datasets are shown in Supplementary Figure 14A.

While our investigation of the dependence of recall on read depth in Figure 2 aimed for a somewhat realistic scenario, we also performed a more controlled experiment in order to see the trends more clearly. We simulated pairs of transcripts with varying relative abundance, edit distance, and read depth (Supplementary Figure 12). The trends show that more reads are needed to recover a transcript when it has lower abundance, when the edit distance to the close transcript is smaller, or when the CCS read quality is lower. Supplementary Figure 12 can be used as a guideline for a prediction of the read depth needed to capture a transcript. A conservative estimate of the total number of reads required to capture a specific transcript can be obtained simply by multiplying the number of reads from the figure by the relative abundance in the figure and dividing by the estimated fraction of the target transcript in the sample. Alternatively, given the read depth of an experiment, Supplementary Figure 12 can give the maximum achievable recall for a transcript based on its abundance and the divergence of its gene family. Finally, we note that the controlled nature of the experiment means that in reality, more reads may be required to achieve the same recall.

Supplementary Note 4: Data processing pipeline

We used a snakemake [13] workflow for the bioinformatic pipeline to process the Iso-Seq data. Supplementary Figure 13 shows the workflow as a graph. The raw data was converted to BAM files using bax2bam, then PacBio’s consensus caller [2] was used to obtain CCS reads from subreads. We used the “classify” algorithm (part of the Tofu pipeline for processing Iso-Seq reads) to separate the CCS reads into reads having at least one full pass and reads shorter than one pass. We further separated the reads having at least one full pass based on the primers into batches using a customized script. We used these primer separated batches of reads for downstream analysis with all tools. That is, ICE, IsoCon and proovread was run on each of these batches separately. Both ICE and IsoCon takes a set of full-pass CCS reads and the base pair quality values and perform clustering and error correction. ICE also uses the reads with less than one full pass in a post-polishing step and we supplied ICE with these reads. ICE further classifies its output transcripts as "high quality" if they have >99% base pair accuracy (default parameter value). We observed that the number of "low quality" transcripts was very high (70-80% of the number of CCS reads) and would give ICE extremely low precision. We therefore used only the high quality transcripts for evaluation.

The consensus caller that generated CCS reads has two modes “--polish” and “--noPolish.” IsoCon and proovread were evaluated on CCS reads generated with the default --polish option, which is the default option. When we evaluated the original PacBio reads we also used CCS reads generated with the --polish option. On the other hand, ICE documentation suggests using reads from the --noPolish option instead. We tried both versions and our experiments confirmed that that the --noPolish option resulted in slightly better metrics in our evaluations of ICE, compared with --polish. ICE with --noPolish predicted 475 high quality transcripts, compared to ICE with --polish, which predicted only 335 high quality transcripts. We observed that ICE with --noPolish had a higher number (9) and percentage (1.9%) of transcripts fully supported by Illumina RNA-seq reads compared to three transcripts (0.9%) with --polish; it had only slightly lower median transcript support of 93.5%, compared to 93.7% with --polish; finally, ICE with --noPolish option also had two more predictions shared between samples (4 compared to 2). ICE with --noPolish however had only 8 exact matches to ENSEMBL compared to 11 for ICE with --polish. It is therefore ambiguous to which input data should be considered giving the best results with ICE, but we conclude that the difference is minor when compared to results of other approaches, irrespective of which dataset we use. Based on these findings and on ICE's documentation, we decided to present the results for ICE with --noPolish reads in the paper.

Finally, we note that for simulated data, we did not run the polishing step. It is not applicable, as we did not generate any base call quality values or non-full-length reads (Sup. Note B).

We provide the exact run-commands used below:

Ccs

```
ccs --numThreads=64 --polish --minLength=10 --minPasses=1 --minZScore=-999 --maxDropFraction=0.8 --minPredictedAccuracy=0.8 --minSnr=4 {input.bam_subreads} {output.ccs_bam}
```

We ran version ccs 2.0.5 (GitHub commit 390a42e), part of the PacBio SMRTlink 4.0 suite. As described above, we also specified the flag --noPolish to generate non-polished reads.

Classify

```
pbtranscript classify  --flnc {output.flnc} --nfl {output.nfl} -d {out} --cpus 64 --min_seq_len 30 -p {targeted_primers} --ignore_polyA {input.ccs_bam} {draft_file}
```

We ran version 1.0.0.177900 of `pbtranscript`, part of the PacBio SMRTlink 4.0 suite.

ICE

```
pbtranscript cluster --quiver  --nfl_fa {input.nfl_by_primer} --bas_fofn {input.bas_fofn}  --blasr_nproc 64 --quiver_nproc 64 --max_sge_jobs 64 -d {out_folder} {input.flnc_by_primer} {output.consensus_transcripts}
```

We ran version 1.0.0.177900 of `pbtranscript`, part of the PacBio SMRTlink 4.0 suite. We initially tried running ICE using the the --targeted_isoseq option but the program stopped with a runtime error, a bug that is not fixed to date (see https://github.com/PacificBiosciences/IsoSeq_SA3nUP/issues/16)

IsoCon

```
./IsoCon pipeline -fl_reads {reads} -outfolder {out_folder} --ccs {ccs_reads.bam}
```

The results of isocon was obtained with commit 79589f3 on GitHub, which is version 0.2.4 of IsoCon. IsoCon predicted transcripts are also included in Sup. Data.

proovread

```
proovread -l {CCS_reads} -s {illumina1} -s {illumina2} -p
{tmp_out} -t  64  --overwrite --no-sampling
```

We ran version 2.14.0 of proovread. We used the --no_sampling option as suggested by the software author, see https://github.com/BioInf-Wuerzburg/proovread/issues/88).

Supplementary Note 5: Analysis of human testes data

Comparing against database: We downloaded coding DNA sequences (CDS) for our 9 ampliconic gene families from the Ensembl database. There were 61 unique sequences in this database. We aligned the predicted transcripts to the Ensembl databases. We consider a perfect match of a transcript to a CDS if the read is a perfect substring of the CDS transcript with at most 100bp missing in the 3’ and 5’ ends. We allow the missing ends because primers in our targeted approach will attach within the start and end exon of a transcript, resulting in missing ends (we observed values in the range of ~20-70 bp). If a predicted transcript has more than one perfect match, we chose the match with the smallest sum of clipped bases in the ends. In case there are several perfect matches with the same number of clipped bases, we pick the lexicographically smallest accession as the match, in order to assign a prediction to a unique transcript in the database.

Support from Illumina reads: We aligned our barcode-separated Illumina reads to the predicted transcripts from IsoCon, ICE and the original CCS reads. We used BWA-MEM with default mapping parameters. We then used a customized script available at https://github.com/ksahlin/IsoCon_Eval/blob/master/analysis/nucleotide_level/get_unsupported_positions.py to count positions on the predicted transcripts that have at least two Illumina reads supporting the base pair. As IsoCon, ICE, original, and Illumina-corrected CCS reads have different number of predicted transcripts for each gene family, the Illumina coverage per transcript is highly different between the four approaches (especially for TSPY and RBMY). Therefore, the coverage per transcript would be lower for original and Illumina corrected reads, thus favoring ICE and IsoCon in the evaluation. To account for this artifact, we split each method's predictions into batches of 50 transcripts. We then align all Illumina reads to each batch separately. This makes Illumina coverage constant across methods and removes any bias resulting from coverage differences.

Number of groups computation: To compute the number of groups, we implemented following post processing algorithm to separate the transcripts into groups (i.e. maximal cliques):

1. Perform Smith-Waterman alignments between all pairs of transcripts in each family. We used SW parameters of *gap_open = -50*, *gap_extend = 0*, *mismatch = -20*, and *match = 1*.
2. Create graph where a node represents an isoform and an edge means that two isoforms can potentially come from the same copy (i.e., no substitution or indel

smaller than 3 base pairs in the pairwise alignment). Graphs are included in Sup. Data.
3. Enumerate all maximal cliques in the graph, using a brute-force algorithm.
4. For each maximal clique, use the pairwise alignments to do a progressive multiple alignment of all the transcripts in the clique. Then derive the consensus sequence. This will contain the full set of exons from all isoforms assigned to a copy. Alignments are included in Sup. Data.

In the case of RBMY transcripts 45-61 (Fig. 5), a manual inspection of the alignments revealed incorrect pairwise alignments, due to short repeats. We tweaked these manually and redid steps 2-4.

Open reading frame (ORF) prediction: Each predicted transcript were aligned to the reference sequences used to design the primers (alignments included in Sup. Data). Those that fully aligned to the references and could be translated into proteins without premature stop codons are categorized as coding; those with premature stop codons are categorized as non-coding. In the case that no stop codon is found, they are categorized as coding. This is because stop codons may be downstream of the primers, or clipped out as part of the primer.

Creating a database of known splice variants: We first constructed a database of known potential splice variants, against which we would later compare IsoCon transcripts. We downloaded all the 105 transcripts of the nine ampliconic gene families from the NCBI RefSeq database [14]. We combined these with the 61 unique CDS sequences from the Ensembl database to obtain 166 known transcripts. We used BLAT (server version, [15]) to align these transcripts to the hg19 reference genome. We filtered out all non chrY alignments. For each transcript, we then considered the highest scoring alignment and any other alignments that had more than 99% identity and 99% coverage (due to the highly repetitive nature of these families). For every alignment, an hg19 deletion of at least 10nt is marked as an intron and its coordinates as splice junction (we varied the 10nt parameter to 5nt and 3nt in this analysis and obtained the same results). In this manner, the 166 known transcripts had 668 distinct candidate splice variants (i.e. a combination of splice junctions). This number is an overestimate but gives us confidence in the novelty of any splice variants we discover that do not match one of the known candidate splice variants. The alignments contained a total of 471 distinct splice junction coordinates which we stored in a database of known potential splice junctions (Supplementary Dataset 1).

Splice variant analysis: To analyze splice variants in IsoCon's transcripts, we aligned them to hg19 and stored the splicing coordinates for the highest scoring alignment and

any other alignment to chrY with >99% identity and >99% coverage. We aimed to classify IsoCon's predictions into three categories: splice match (SM), novel in catalog (NIC) and "ambiguous" (part of terminology used in [16]). SM transcripts are transcripts that have identical splice coordinates to a reference transcript in all its junctions. The NIC transcripts are transcripts that "contain new combinations of already annotated splice junctions or novel splice junctions formed from already annotated donors and acceptors " [16] However, these classifications rely on accurate alignments which, given the repetitive nature of our gene families and that the reference does not contain all gene copies, are not always obtainable. We therefore classify transcripts without clear SM or NIC alignments as ambiguous, as they might either contain novel splice junction(s) or be novel distinct gene copies (i.e., not having a representation of the reference genome). In the case of transcripts with more than one alignment, we did not see mixed categories, i.e. all the alignments were either SC, NIC, or ambiguous. We also note that for NIC transcripts, we cannot with full confidence distinguish if it is a true novel splice variant of an existing gene copy or if the transcript is a novel distinct gene copy itself. For IsoCon's 121 predictions that were shared between samples, we found that 83 transcripts were classified as SM, 21 transcripts as NIC, 17 as ambiguous (NIC and ambiguous are presented in Supplementary Table 1).

Analysis of transcripts with lower significance: For each transcript, IsoCon outputs a significance value which is the maximum of all its pairwise significance values with other transcripts. We investigated the possibility that transcripts with lower significance have an enrichment of false positives. To do this, we investigated our accuracy metrics as a function of significance values, focusing on transcripts with a value greater than 10e-20 (Supplementary Figure 15). We observe that the percentage of transcripts with a p-value greater than 10e-20 that have full Illumina support is 67%. This is lower than the 80% for all the transcripts with lower p-values, but still fairly high. For reference, recall that for ICE and proovread these numbers are 2% and 15%, respectively. When looking at percentage of nucleotides supported by Illumina, it is 99.0% for transcripts with a p-value greater than 10e-20, which is almost as high as for transcripts with lower p-values (99.2%). For reference, recall that ICE and proovread transcripts have support of 93% and 96%, respectively. We also observe that out of the 21 distinct transcripts recovered from the Ensemble database, four were recovered by IsoCon predictions with a significance greater than 10e-8. transcripts. This indicates that retaining predictions with higher p-values is likely necessary to obtain higher sensitivity.

Additionally, p-values of all IsoCons predictions are shown in Supplementary Figure 14B.

Running time and memory analysis: We compared runtime and memory of IsoCon, ICE, and proovread on our human samples (Supplementary Table 2). We used a machine with an x86_64 system running Linux v3.2.0-4-amd64 and equipped with 32 2-threaded cores and 512 GB RAM. IsoCon is roughly 2x faster than ICE when running on a single core, and ~5x faster when running over 64 processes. A direct comparison to proovread is challenging because it requires a minimum read length criteria that makes it unable to process the *BPY* and *XKRY* families. With this caveat in mind, proovread run times were 12% slower than IsoCon on 1 core and >3x slower on 64 cores.

Supplementary Note 6: Analysis of FMR1 data

We downloaded the 49 isoforms of the FMR1 gene detected in [17] from https://zenodo.org/record/833502#.Wsav_NPwZTb . We also included the three isoforms that were predicted in a previous study [18] but not found in [17]. These three isoforms were constructed from hg19 based on the splicing coordinates given in [18]. We refer to these 52 as validation isoforms. We used BLAT (server version, [15]) to align these transcripts to the hg19 reference genome and stored, for each isoform, the unique set of splicing coordinates on hg19.

The original CCS read data was downloaded in fastq format from (https://zenodo.org/record/185011#.WtEWadPwZTY). We ran IsoCon on each of the six samples individually with the same parameters as for the ampliconic gene families (default settings, see Supplementary Note D). We then aligned all IsoCon's predictions using the server version of BLAT. We then replicated the filtering criteria of [17] -- we retained only the predicted transcripts with an alignment coverage >= 99%, identity >= 95%, and location within the area targeted by the primer design (start between chrX:147,014,079–147,014,470 and end between chrX:147030158-147030505).

We classified each of the 52 validation isoforms as detected if there was a predicted transcript that had identical donor and acceptor splice coordinates across all exons in the transcript. All 52 validation isoforms have distinct splice-junction coordinates, which means that each IsoCon prediction can be assigned to at most one validation isoform.

We ran IsoCon for the FMR1 datasets on a MacBook Pro with three 3.1 GHz Intel Core i7 cores and 16 GB 1867 MHz DDR3. The three premutation carrier samples containing CCS reads from 3 SMRT cells respectively were all processed by IsoCon in approximately 3 hours with a maximum memory consumption of less than a gigabyte. The controls, each of which contained CCS reads from 1 SMRT cell, were each processed in between 30-40 minutes.

Supplementary References

1. Ewing, B. & Green, P. Base-calling of automated sequencer traces using phred. II. Error probabilities. *Genome Res.* **8,** 186–194 (1998).
2. Töpfer, A. PacificBiosciences/unanimity. *GitHub* (2017). Available at: https://github.com/PacificBiosciences/unanimity. (Accessed: 20th December 2017)
3. Šošić, M. & Šikić, M. Edlib: a C/C++ library for fast, exact sequence alignment using edit distance. *Bioinformatics* (2017). doi:10.1093/bioinformatics/btw753
4. Kersey, P. J. *et al.* Ensembl Genomes 2018: an integrated omics infrastructure for non-vertebrate species. *Nucleic Acids Res.* (2017). doi:10.1093/nar/gkx1011
5. Ono, Y., Asai, K. & Hamada, M. PBSIM: PacBio reads simulator--toward accurate genome assembly. *Bioinformatics* **29,** 119–121 (2013).
6. Lau, B. *et al.* LongISLND: in silico sequencing of lengthy and noisy datatypes. *Bioinformatics* **32,** 3829–3832 (2016).
7. Baker, E. A. G., Goodwin, S., Richard McCombie, W. & Ramos, O. M. SiLiCO: A Simulator of Long Read Sequencing in PacBio and Oxford Nanopore. (2016). doi:10.1101/076901
8. Kotz, S. & Van Dorp, J. R. *Beyond Beta: Other Continuous Families of Distributions with Bounded Support and Applications*. (World Scientific, 2004).
9. Rhoads, A. & Au, K. F. PacBio Sequencing and Its Applications. *Genomics Proteomics Bioinformatics* **13,** 278–289 (2015).
10. PacBio. Specifics of SMRT Sequencing Data. *Speaker Deck* (2013). Available at: https://speakerdeck.com/pacbio/specifics-of-smrt-sequencing-data. (Accessed: 16th November 2017)
11. Laehnemann, D., Borkhardt, A. & McHardy, A. C. Denoising DNA deep sequencing data—high-throughput sequencing errors and their correction. *Brief. Bioinform.* **17,** 154–179

(2015).

12. Carneiro, M. O. *et al.* Pacific biosciences sequencing technology for genotyping and variation discovery in human data. *BMC Genomics* **13,** 375 (2012).

13. Köster, J. & Rahmann, S. Snakemake--a scalable bioinformatics workflow engine. *Bioinformatics* **28,** 2520–2522 (2012).

14. National Center for Biotechnology Information (NCBI). in *The Dictionary of Genomics, Transcriptomics and Proteomics* 1–1 (2015).

15. Kent, W. J. BLAT--the BLAST-like alignment tool. *Genome Res.* **12,** 656–664 (2002).

16. Tardaguila, M. *et al.* SQANTI: extensive characterization of long read transcript sequences for quality control in full-length transcriptome identification and quantification. (2017). doi:10.1101/118083

17. Tseng, E., Tang, H.-T., AlOlaby, R. R., Hickey, L. & Tassone, F. Altered expression of the FMR1 splicing variants landscape in premutation carriers. *Biochim. Biophys. Acta* **1860,** 1117–1126 (2017).

18. Pretto, D. I. *et al.* Differential increases of specific FMR1 mRNA isoforms in premutation carriers. *J. Med. Genet.* **52,** 42–52 (2015).

19. Tseng, E., Tang, H.-T., AlOlaby, R. R., Hickey, L. & Tassone, F. Altered expression of the FMR1 splicing variants landscape in premutation carriers. *Biochim. Biophys. Acta* **1860,** 1117–1126 (2017).

20. Pretto, D. I. *et al.* Differential increases of specific *FMR1* mRNA isoforms in premutation carriers. *J. Med. Genet.* **52,** 42–52 (2015).

21. Töpfer, A. PacificBiosciences/unanimity. *GitHub* (2017). Available at: https://github.com/PacificBiosciences/unanimity. (Accessed: 20th December 2017)

Supplementary Tables

Supplementary Table 1. Novel splice variants. The table shows all transcripts that do not have an exact splice match to a reference database transcript. They are classified as either “novel-in-catalog” (NIC) if they “contain new combinations of already annotated splice junctions or novel splice junctions formed from already annotated donors and acceptors” [59], or "ambiguous” if they have at least one splice junction not matching any reference transcript. The column splice-variant-id shows which splice variant group the transcript belongs to after grouping the transcripts based on their splice coordinates, i.e. two transcripts with the same id only differ within exons.

accession number in sample 1	family	splice variant id	category	CCS read support in sample 1	protein-coding	Illumina support
transcript_49_support_9_9_not_tested_9_	DAZ	0	ambiguous	9	no	full
transcript_198_support_7_11_3.0499935358348083e-12_15_S	TSPY	1	ambiguous	11	yes	full
transcript_241_support_2_2_2.465494800796927e-27_5_SSSSSS	RBMY	2	NIC	2	yes	full
transcript_410_support_16_15_7.158588427719199e-51_23_DDDDD	RBMY	3	NIC	15	no	full
transcript_33_support_4_3_1.17730715237844e-20_6_SSS	DAZ	4	ambiguous	3	yes	full
transcript_39_support_27_41_2.989824769295383e-97_45_S	DAZ	5	ambiguous	41	yes	full
transcript_411_support_20_16_4.4071493360773237e-60_25_SI	RBMY	6	NIC	16	no	full
transcript_194_support_3_3_not_tested_3_	TSPY	7	ambiguous	3	no	full
transcript_250_support_18_29_1.8734940341406434e-68_34_S	RBMY	8	NIC	29	no	full
transcript_448_support_9_27_1.8747539629510054e-18_369_DD	RBMY	9	ambiguous	27	no	candidate junction not supported
transcript_7_support_12_11_2.8055256364539264e-30_14_S	DAZ	10	ambiguous	11	yes	full
transcript_185_support_2_2_0.0002504605210692139_9_S	TSPY	11	ambiguous	2	no	candidate junction not supported
transcript_29_support_111_178_3.2981431164247527e-187_222_I	DAZ	12	ambiguous	178	yes	full
transcript_6_support_7_7_not_tested_7_	PRY	13	ambiguous	7	no	full

transcript_4_support_80_78_1.6878832644605026e-253_83_S	PRY	14	ambiguous	78	no	full
transcript_35_support_6_8_not_tested_8_	RBMY	15	NIC	8	no	full
transcript_35_support_6_6_not_tested_6_	TSPY	16	ambiguous	6	yes	full
transcript_409_support_6_5_5.611166030863804e-37_12_SSS	RBMY	17	NIC	5	no	full
transcript_474_support_151_213_not_tested_213_	RBMY	18	NIC	213	no	full
transcript_475_support_14_16_2.7853968732079244e-19_229_S	RBMY	18	NIC	16	no	full
transcript_236_support_3_3_2.111151999517751e-08_7_S	TSPY	19	ambiguous	3	yes	full
transcript_136_support_4_4_1.5730541529242116e-13_7_S	TSPY	19	ambiguous	4	yes	full
transcript_319_support_10_3_1.0769783944960056e-08_8_S	RBMY	20	NIC	3	yes	full
transcript_240_support_4_5_1.4154227615021973e-12_8_S	RBMY	20	NIC	5	yes	full
transcript_29_support_3_3_2.149596591708188e-09_6_S	TSPY	21	ambiguous	3	yes	full
transcript_28_support_3_3_7.906644316104741e-07_6_S	TSPY	21	ambiguous	3	yes	full
transcript_110_support_13_13_2.0722759276211275e-48_21_SS	RBMY	22	NIC	13	no	full
transcript_109_support_4_6_2.593261298336897e-30_21_SS	RBMY	22	NIC	6	no	full
transcript_413_support_8_11_5.398149132019713e-30_14_S	RBMY	23	NIC	11	no	full
transcript_412_support_2_3_8.987210687229348e-08_14_S	RBMY	23	NIC	3	no	full
transcript_246_support_23_12_2.0292740496871315e-23_27_S	RBMY	24	NIC	12	no	full
transcript_129_support_11_10_1.5336157152280373e-14_30_S	RBMY	24	NIC	10	no	full
transcript_107_support_7_9_4.1466952828840725e-37_39_SS	RBMY	25	NIC	9	no	full
transcript_108_support_22_28_1.8160411545666335e-74_35_S	RBMY	25	NIC	28	no	full
transcript_97_support_8_6_1.2825651250976259e-14_10_S	TSPY	26	NIC	6	no	full
transcript_91_support_3_3_9.201510164493637e-08_10_S	TSPY	26	NIC	3	no	full
transcript_208_support_4_4_2.003323587591366e-10_8_S	TSPY	26	NIC	4	no	full
transcript_2_support_40_52_not_tested_52_	XKRY*	27	ambiguous	52	yes	full

Supplementary Table 2. Runtime and peak memory usage of IsoCon, ICE, and proovread to process the whole biological dataset. Note that proovread did not perform any correction of reads from the *BPY* and *XKRY* families, due to their short size.

	IsoCon		ICE		proovread	
	1 thread	64 threads	1 thread	64 threads	1 thread	64 threads
Wall clock time (hh:mm)	08:02	00:41	15:52	03:32	9:02*	2:07*
Peak memory (GB)	0.72	0.73	1.75	1.77	4.57*	5.85*

Supplementary Table 3. Absence or presence of our *FMR1* validation set of 52 isoforms from [19,20], as detected by IsoCon in each sample. We use the group and isoform nomenclature to match [19,20]). The numbers indicate the total number of CCS reads supporting the isoform, and a dash indicates that the isoform was not found in the sample. As IsoCon can predict several transcripts with the same isoform structure (i.e. differing only in mutations), the supporting reads are collected from all the matching transcripts.

Group	Isoform/ PB#	Carrier-1	Carrier-2	Carrier-3	Normal-1	Normal-2	Normal-3
A	Isoform 1	182	46	60	-	12	7
A	Isoform 2	55	6	22	-	-	-
A	Isoform 3	67	13	40	3	11	-
A	Isoform 13	190	57	232	8	17	68
A	Isoform 14	39	17	12	3	-	18
A	Isoform 15	74	41	64	-	4	9
B	Isoform 4b	17	6	-	-	-	-
B	Isoform 6	62	125	11	-	-	-
B	Isoform 16	23	10	59	-	-	-
C	Isoform 7	4808	3236	4109	439	506	772
C	Isoform 8	817	451	775	91	111	273
C	Isoform 9	1113	736	1018	54	72	166
C	Isoform 17	4894	5033	4785	819	885	1356
C	Isoform 18	560	1721	972	227	131	571
C	Isoform 19	901	1043	1217	60	87	499
D	Isoform 10	54	116	167	11	10	-
D	Isoform 10b	192	28	229	12	9	-
D	Isoform 11	117	-	38	-	4	-
D	Isoform 11b	88	82	170	3	7	-
D	Isoform 12	265	48	86	11	30	4
D	Isoform 20	68	52	454	25	87	240
E	PB.1.22	-	-	3571	-	-	-
E	PB.1.23	-	-	33	-	-	-
E	PB.1.24	9	181	-	-	-	-
E	PB.1.25	13	4936	5	-	-	7
E	PB.1.32	-	-	1284	-	-	-
E	PB.1.35	-	-	-	-	-	-
E	PB.1.44	-	158	-	-	-	6
E	PB.1.45	-	135	-	-	-	-
E	PB.1.56	-	18	-	-	-	-
E	PB.1.57	-	-	122	-	-	3
F	PB.1.13	-	21	-	-	-	-
F	PB.1.14	117	-	-	-	-	-
F	PB.1.21	-	29	6	-	-	-
F	PB.1.26	22	-	-	-	-	-

F	PB.1.29	-	-	30	-	-	-
F	PB.1.30	-	-	56	-	-	-
F	PB.1.31	-	28	-	-	-	-
F	PB.1.33	-	59	-	-	-	-
F	PB.1.34	6	-	27	-	-	-
F	PB.1.36	-	59	-	-	-	-
F	PB.1.39	-	95	-	-	-	-
F	PB.1.41	-	-	-	-	-	12
F	PB.1.42	-	195	-	-	-	-
F	PB.1.47	-	-	4	-	-	8
F	PB.1.50	4	49	-	-	4	-
F	PB.1.54	-	19	-	-	-	-
F	PB.1.55	-	-	135	-	-	-
F	PB.1.9	-	-	-	96	55	20
B (Pretto 2015)	Isoform_4	-	-	5	-	-	-
B (Pretto 2015)	Isoform_5	-	-	-	-	-	-
B (Pretto 2015)	Isoform_5b	-	6	-	-	-	-
Total isoforms detected in sample		27	35	32	15	17	18

Supplementary Table 4. RT-PCR primers designed in the first and last coding exons of the nine Y ampliconic gene families. Each primer starts with a 21 bp-long PacBio barcode that is unique for each sample.

Amplification target	Primer name	Primer sequence
	BPY2_Sam1_F4	GGTAGGCGCTCTGTGTGCAGCCGTGCAGGACAGGATCATTA
BPY_sample1	BPY2_Sam1_R	CCATCTCATATGTAGTACTCTTTACTTCTGTGATCTGGGC
	CDY1/2_Sam1_F	GGTAGGCGCTCTGTGTGCAGCTTCCCAGGAGTTTGAGGTTG
CDY1_sample1	CDY1/2_Sam1_R1	CCATCTCATATGTAGTACTCTCTCATCAATTTTATTTTCAACATAC
	CDY1/2_Sam1_F	GGTAGGCGCTCTGTGTGCAGCTTCCCAGGAGTTTGAGGTTG
CDY2_sample1	CDY1/2_Sam1_R2	CCATCTCATATGTAGTACTCTTCAAGGGCACCATCTCTGAT
	DAZ_Sam1_F2	GGTAGGCGCTCTGTGTGCAGCACCACTCGAAGCCCCACA
DAZ_sample1	DAZ_Sam1_R	CCATCTCATATGTAGTACTCTTCTGGATTAAACAGACAAGATACCA
	HSFY_Sam1_F	GGTAGGCGCTCTGTGTGCAGCACTCAAGATGTTTCCCCCAAA
HSFY_sample1	HSFY1_Sam1_R	CCATCTCATATGTAGTACTCTTTGTCCAGTGGTGATGGTTG
	PRY_Sam1_F1	GGTAGGCGCTCTGTGTGCAGCATGGGAGCCACTGGGCTTG
PRY_sample1	PRY_Sam1_R	CCATCTCATATGTAGTACTCTCACAGATGTCCCCAAGTGC
	RBMY_Sam1_F	GGTAGGCGCTCTGTGTGCAGCAGCAGATCATCCTGGCAAGC
RBMY_sample1	RBMY_Sam1_R	CCATCTCATATGTAGTACTCTTTAATATCTGCTCGAGTCTCCTTTT
	TSPY_Sam1_F	GGTAGGCGCTCTGTGTGCAGCAGGGCTCGCTGACCTAC
TSPY_sample1	TSPY_Sam1_R2	CCATCTCATATGTAGTACTCTTCAACTCAACAACTGGGAGTC
	VCY_Sam1_F	GGTAGGCGCTCTGTGTGCAGCATGAGTCCAAAGCCGAGAGC
VCY_sample1	VCY_Sam1_R	CCATCTCATATGTAGTACTCTTCAGGGAGATAGGGGAGTAGA
	XKRY_Sam1_F	GGTAGGCGCTCTGTGTGCAGCTTAATAGCATTGCTGATGACATATTCC
XKRY_sample1	XKRY_Sam1_R	CCATCTCATATGTAGTACTCTGATAAGCATCCATACTTACCCACCA
	BPY2_Sam2_F4	GGTAGTCATGAGTCGACACTACGTGCAGGACAGGATCATTA
BPY_sample2	BPY2_Sam2_R	CCATCGCGATCTATGCACACGTTACTTCTGTGATCTGGGC
	CDY1/2_Sam2_F	GGTAGTCATGAGTCGACACTATTCCCAGGAGTTTGAGGTTG
CDY1_sample2	CDY1/2_Sam2_R1	CCATCGCGATCTATGCACACGCTCATCAATTTTATTTTCAACATAC
	CDY1/2_Sam2_F	GGTAGTCATGAGTCGACACTATTCCCAGGAGTTTGAGGTTG
CDY2_sample2	CDY1/2_Sam2_R2	CCATCGCGATCTATGCACACGTCAAGGGCACCATCTCTGAT
	DAZ_Sam2_F2	GGTAGTCATGAGTCGACACTAACCACTCGAAGCCCCACA
DAZ_sample2	DAZ_Sam2_R	CCATCGCGATCTATGCACACGTCTGGATTAAACAGACAAGATACCA
	HSFY_Sam2_F	GGTAGTCATGAGTCGACACTAACTCAAGATGTTTCCCCCAAA
HSFY_sample2	HSFY1_Sam2_R	CCATCGCGATCTATGCACACGTTGTCCAGTGGTGATGGTTG
	PRY_Sam2_F1	GGTAGTCATGAGTCGACACTAATGGGAGCCACTGGGCTTG
PRY_sample2	PRY_Sam2_R	CCATCGCGATCTATGCACACGCACAGATGTCCCCAAGTGC
	RBMY_Sam2_F	GGTAGTCATGAGTCGACACTAAGCAGATCATCCTGGCAAGC
RBMY_sample2	RBMY_Sam2_R	CCATCGCGATCTATGCACACGTTAATATCTGCTCGAGTCTCCTTTT
	TSPY_Sam2_F	GGTAGTCATGAGTCGACACTAAGGGCTCGCTGACCTAC
TSPY_sample2	TSPY_Sam2_R2	CCATCGCGATCTATGCACACGTCAACTCAACAACTGGGAGTC
	VCY_Sam2_F	GGTAGTCATGAGTCGACACTAATGAGTCCAAAGCCGAGAGC
VCY_sample2	VCY_Sam2_R	CCATCGCGATCTATGCACACGTCAGGGAGATAGGGGAGTAGA
	XKRY_Sam2_F	GGTAGTCATGAGTCGACACTATTAATAGCATTGCTGATGACATATTCC
XKRY_sample2	XKRY_Sam2_R	CCATCGCGATCTATGCACACGGATAAGCATCCATACTTACCCACCA

Supplementary Figures

Panel A

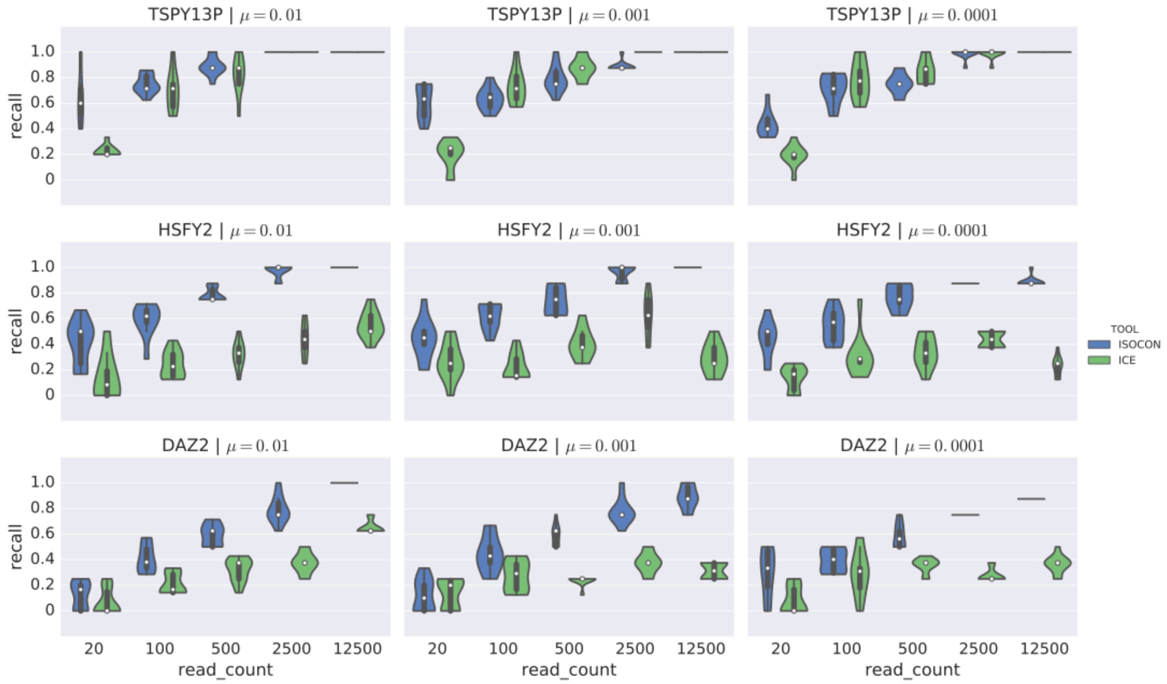

Panel B

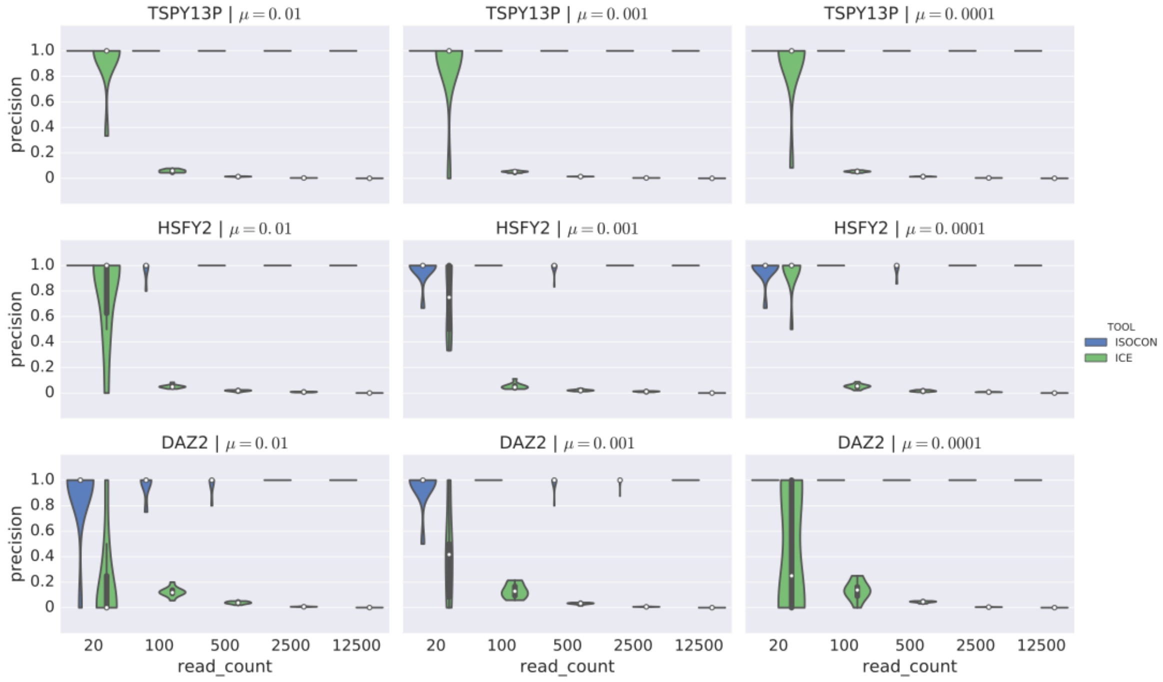

Supplementary Figure 1. Recall and precision on simulated families of transcripts with the same exon structure and unequal abundance rates. Violin plots showing the recall (panel A) and precision (panel B) of IsoCon and ICE.

Each plot shows results for a total of 8 transcripts with abundances randomly assigned and ranging from 0.4% to 50%.

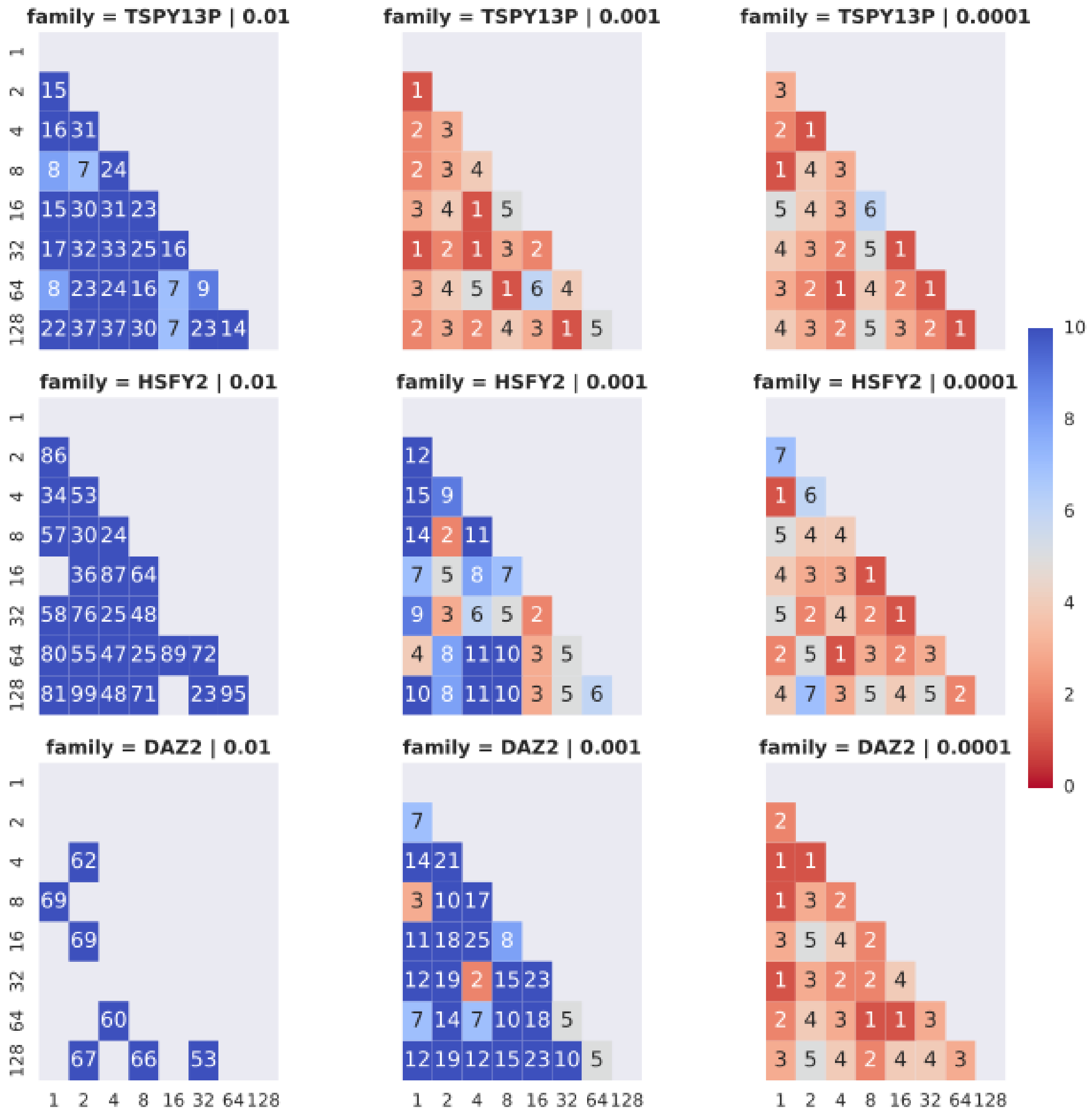

Supplementary Figure 2. Edit distance between gene copies for simulated datasets of 8 gene copies. Each matrix shows edit distances between the 8 simulated copies for a specific gene family and mutation rate. The x and y axis show the abundance level of the copy in the unequal abundance experiment. The number in each block shows the edit distance between copies. Numbers on or above the diagonal are masked. Also masked are any blocks with edit distance >99.

Panel A

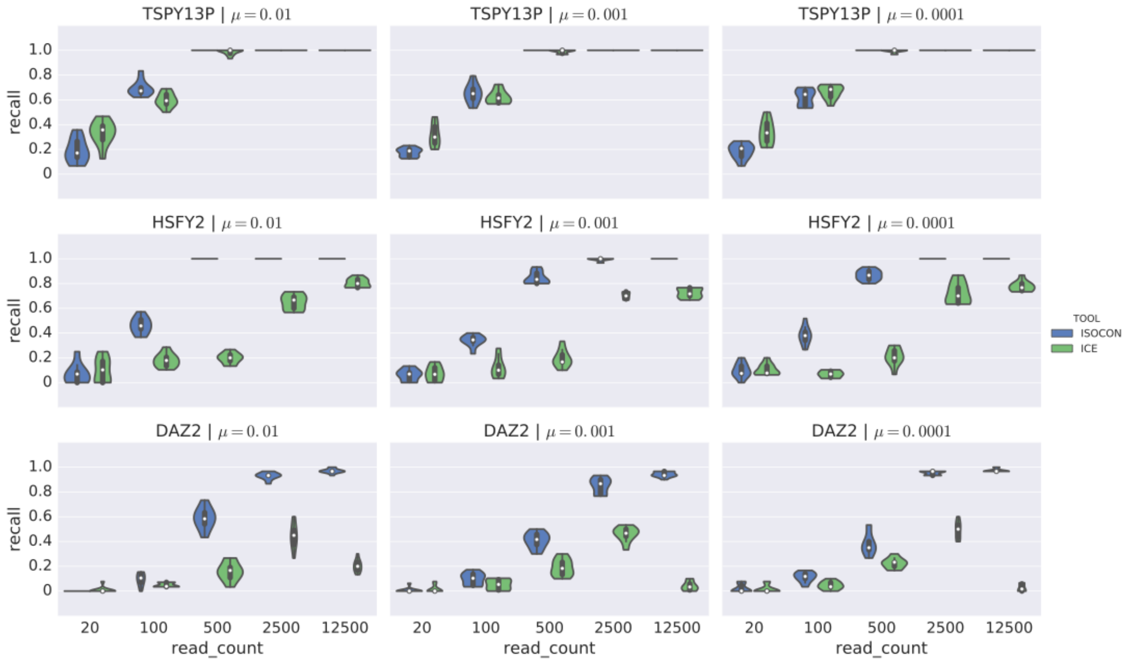

Panel B

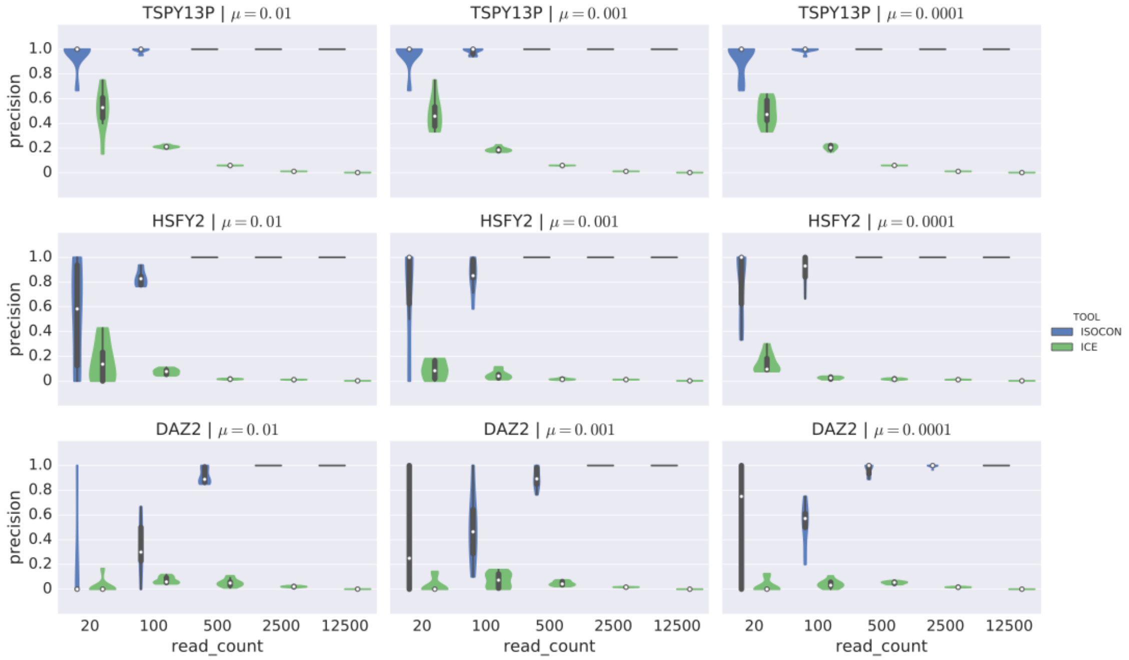

Supplementary Figure 3. Violin plots showing the recall (panel A) and precision (panel B) of IsoCon and ICE on simulated families of transcripts with different exon structure and equal abundance rates. Each plot shows results for a total of 30 isoforms with equal abundances.

Panel A

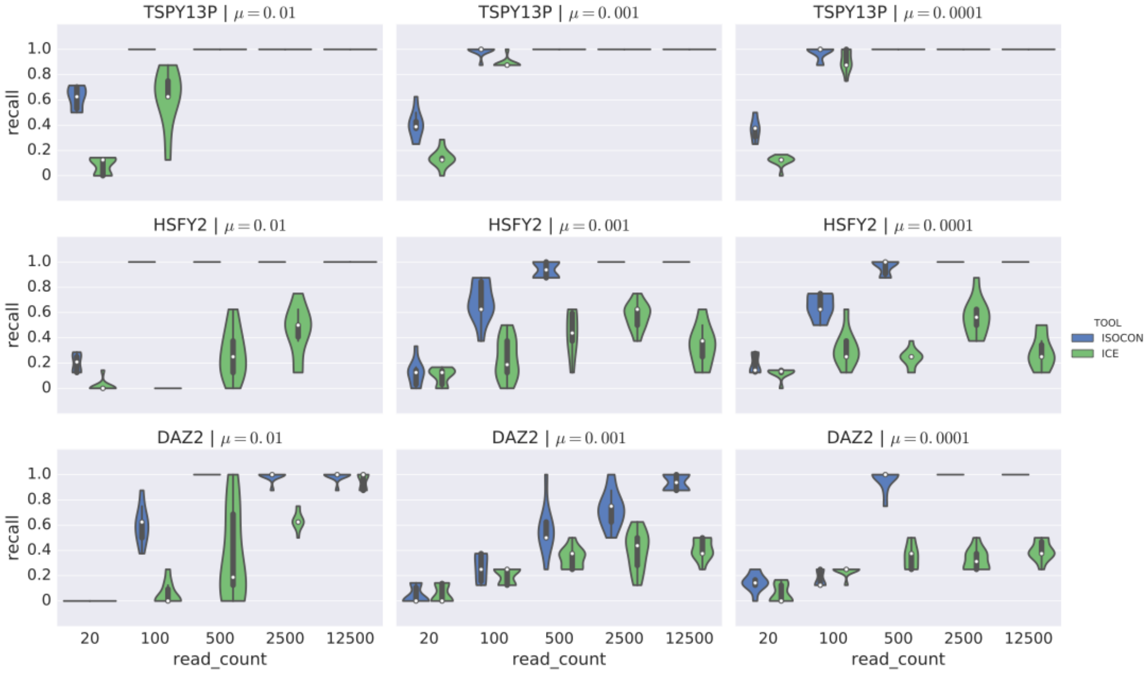

Panel B

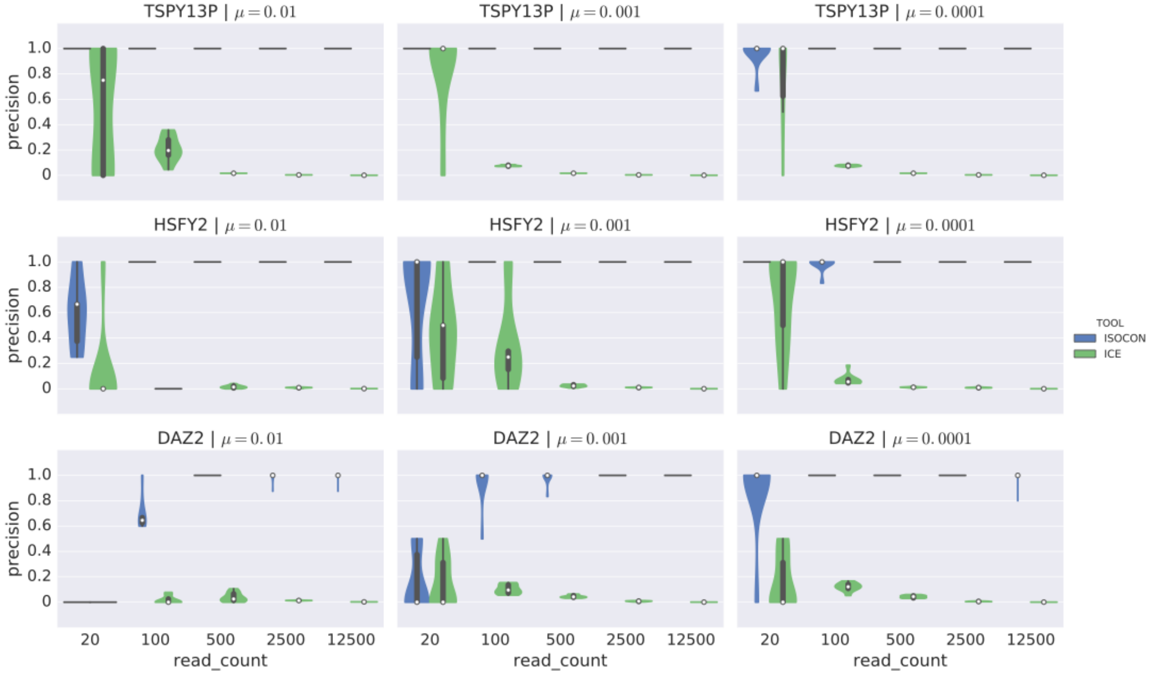

Supplementary Figure 4. Violin plots showing the recall (panel A) and precision (panel B) of IsoCon and ICE on simulated families of transcripts with the same exon structure and equal abundance rates. Each plot shows results for a total of 8 transcripts.

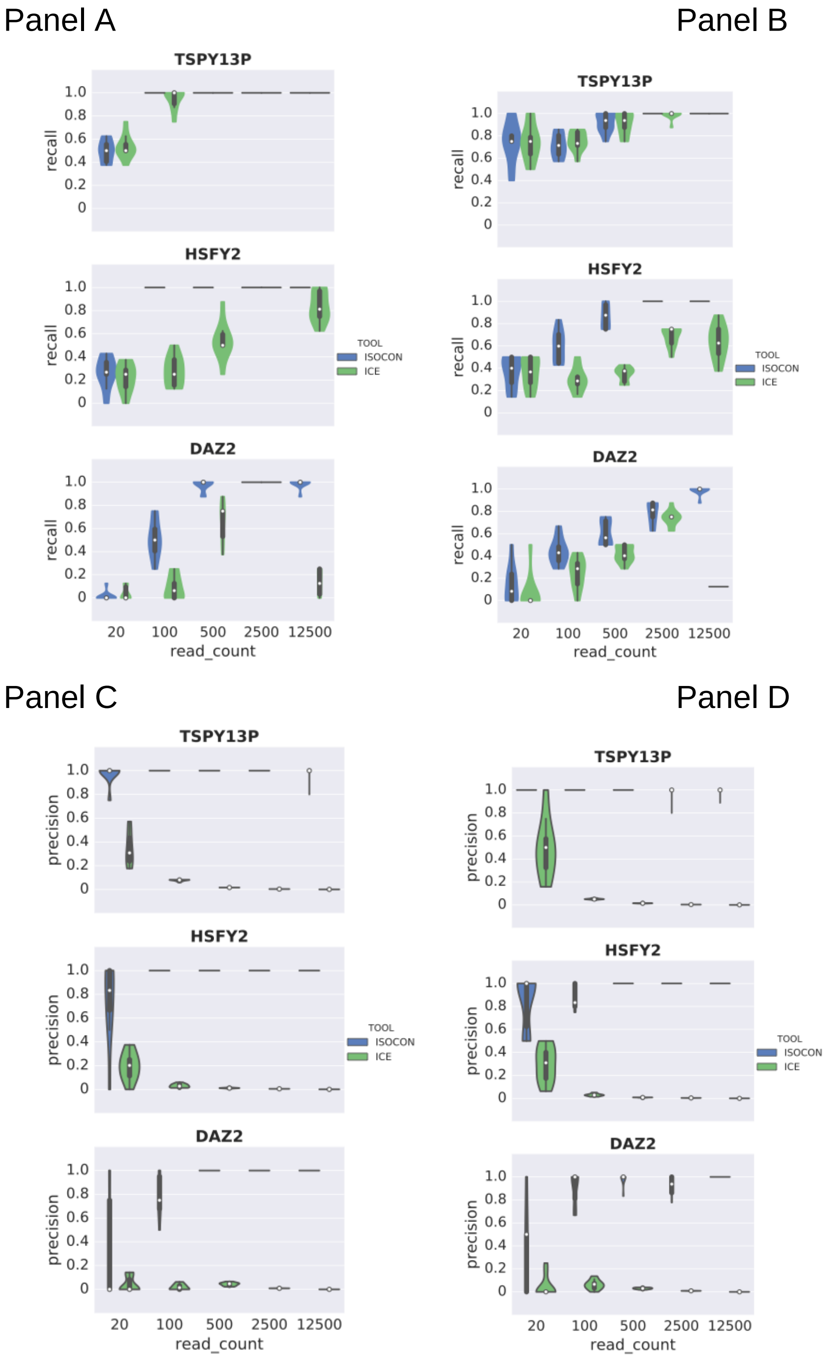

Supplementary Figure 5. Violin plots showing the recall (panel A-B) and precision (panel C-D) of IsoCon and ICE on a single gene copy with eight different isoforms with equal abundance rates (left) and unequal abundance rates (right).

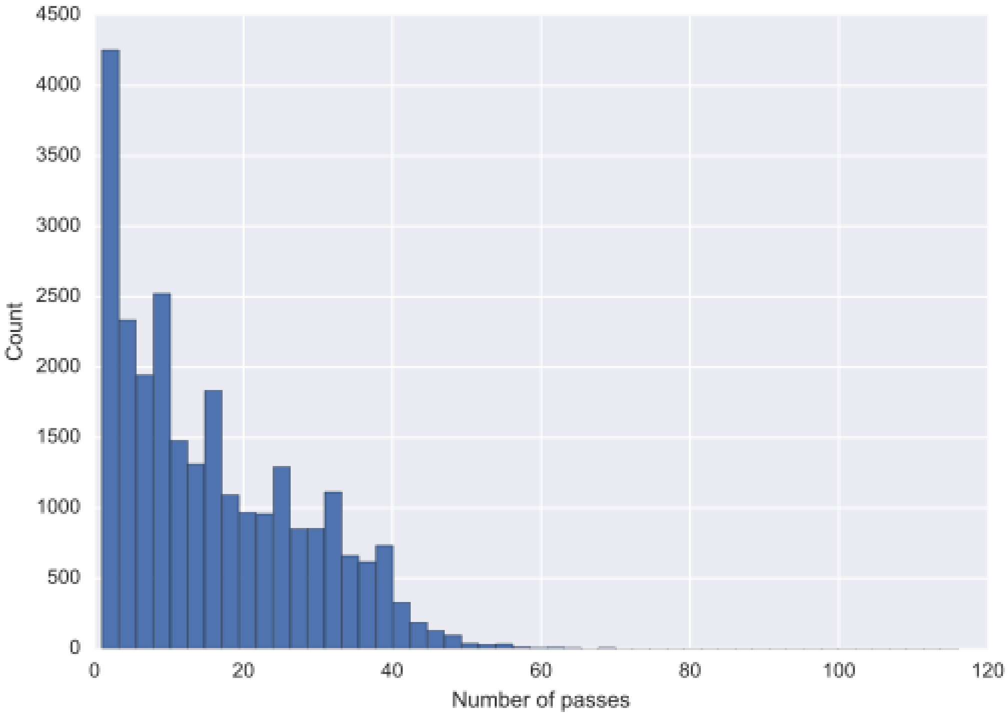

Supplementary Figure 6. Histogram showing the number of polymerase passes in the CCS reads for both samples**.** The average number of passes is 16 and the median is 13.

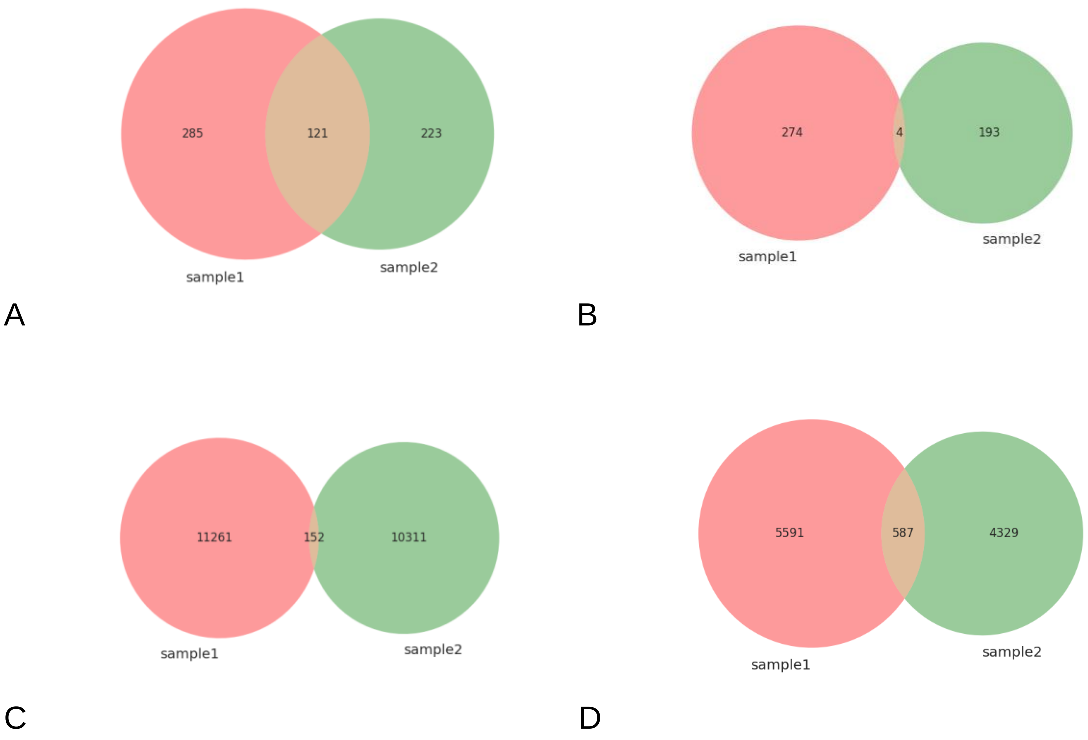

Supplementary Figure 7. Venn diagram of the number of predicted transcripts shared between two samples. Panel (A) IsoCon, (B) ICE, (C) original reads, and (D) Illumina corrected CCS reads. A transcript is shared if it has a perfect match between samples (edit distance of 0). Identical sequences within one sample are collapsed.

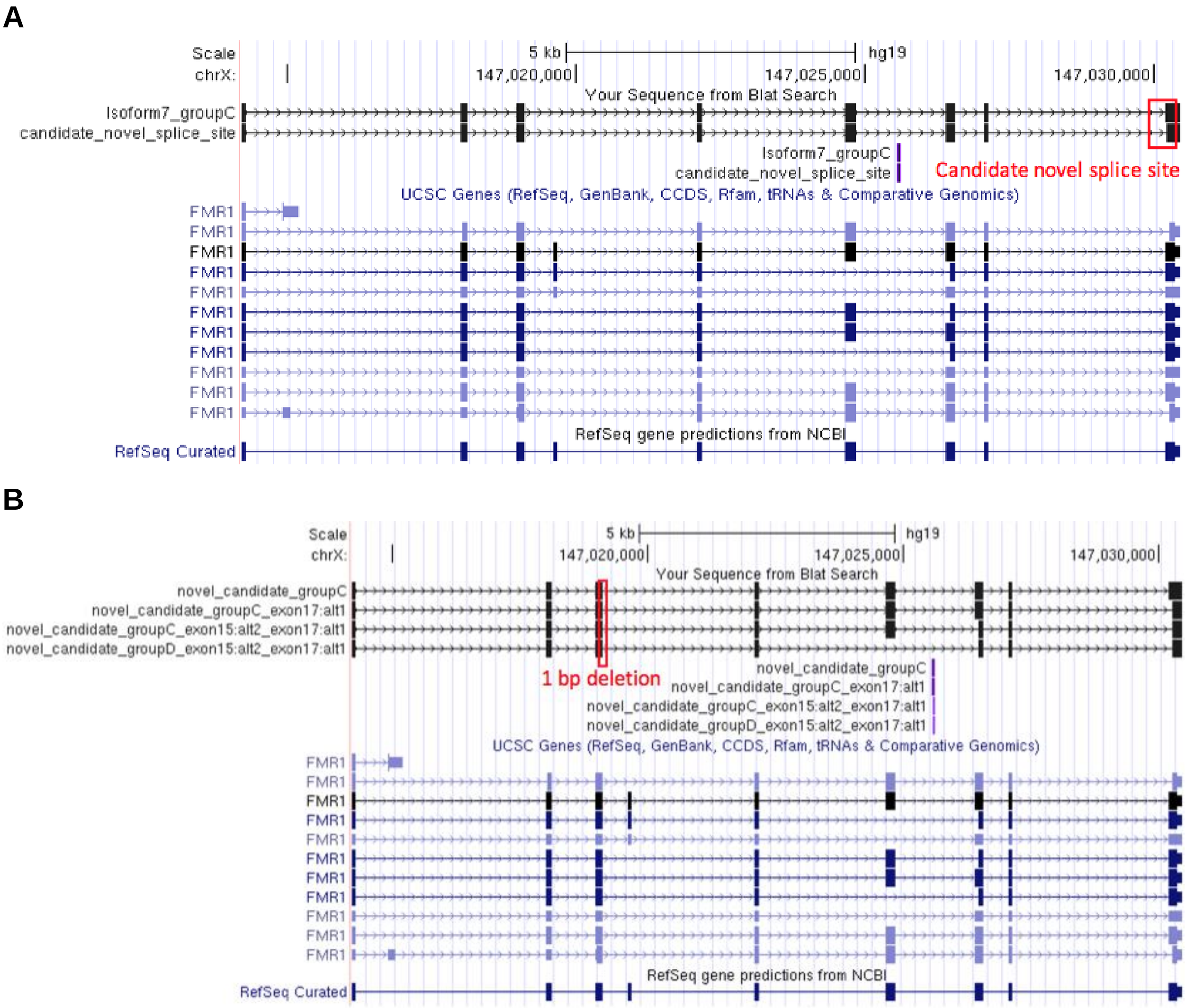

Supplementary Figure 8. (A) The alignment of two transcripts in the *FMR1* dataset (exon 9-17; the targeted region in this dataset). Aligned sequence Isoform7_groupC is a previously reported transcript and candidate_novel_splice_site is a transcript derived by IsoCon. The candidate_novel_splice_site transcript has a novel splice site occuring 5bp downstream of the RefSeq curated exon 17. Alignments of other known UCSC isoforms are also shown. **(B)** Four IsoCon detected isoforms with the 1bp deletion at the end of exon 11 forming a candidate for a new non-canonical splice site. The isoforms differ only in this deletion to the closest previously annotated isoform. We confirmed that this variant occurred in at least 2486 CCS reads in each of the premutation samples (which is over 10% of the reads), and in at least 370 CCS reads in each of the control samples (which is over 3% of the reads).

PartitionStrings$(\mathcal{S})$

Input: A set of strings $\mathcal{S}$.

Output: A partition of $\mathcal{S}$ into ℓ clusters $S_1, \ldots, S_\ell$, and designated central elements $c_1, \ldots, c_\ell$.

Let G be the nearest neighbor graph of $\mathcal{S}$

Set $\ell = 0$. `// Number of clusters created`

while G *is non-empty* **do**

  Let s be a read with the most nodes that can reach s in G.

  Let R be the reads from which s is reachable in G.

  Let $\ell = \ell + 1$. `// Create a new cluster`

  Set $S_\ell = \{s\} \cup R$.

  Set $c_\ell = s$.

  Remove S_ℓ from G.

end

return $S_1, \ldots, S_\ell$ and $c_1, \ldots, c_\ell$.

Algorithm 1: PartitionStrings routine

ClusterCorrect$(\mathcal{S})$

Input: $\mathcal{S}$ — a set of strings which are the Pacbio reads

Output: a collection of strings that are candidate transcripts.

$S_1, \ldots, S_\ell, c_1, \ldots, c_\ell = \text{PartitionStrings}(\mathcal{S})$.

while *not all parts are converged* **do**

  for *each non-converged cluster* S_i **do**

    Compute multiple alignment from reads in S_i and c_i.

    Compute consensus.

    for $s \in S_i$ **do**

      Correct some positions in s, as described in the text.

    end

  end

  $S_1, \ldots, S_\ell, c_1, \ldots, c_\ell = \text{PartitionStrings}(\mathcal{S})$.

end

return $c_1, \ldots, c_\ell$.

Algorithm 2: ClusterCorrect routine

IsoCon$(\mathcal{X})$

Input: $\mathcal{X}, \tau, \alpha$ — $\mathcal{X}$ is a multiset of PacBio reads, τ is the maximum number of insignificant candidates to be removed during an iteration, and α is a significance threshold.

Output: A set of final predicted transcripts.

Let $\mathcal{S} = \mathcal{X}$ `// Make a copy of the reads that we can modify`

Let $\mathcal{C} = \{c_1, \ldots, c_\ell\} = \text{ClusterCorrect}(\mathcal{S})$

do

  Assign reads to candidates as described in the text.

  Let $\mathcal{X}_i$ denote the reads assigned to c_i.

  Let $\rho_1, \ldots, \rho_\ell = 0, \ldots, 0$ `// Initialize storage of significance values.`

  Let G be the nearest neighbor graph of $\mathcal{C}$.

  for *each edge* (c_i, c_j) *in* G **do**

    $\rho = \text{SignificanceTest}(c_i, c_j, X_i, X_j)$

    if $\rho > \rho_i$ **then**

      $\rho_i = \rho$

  end

  Let $\mathcal{D} = \{c_i \in \mathcal{C} \mid \rho_i > \alpha \text{ and } \rho_i \text{ is in the highest } \tau \text{ values of } \{\rho_1, \ldots, \rho_\ell\}\}$

  $\mathcal{C} = \mathcal{C} \setminus \mathcal{D}$

while $\mathcal{D} \neq \emptyset$

return $\mathcal{C}$

Algorithm 3: The IsoCon algorithm

Supplementary Figure 9. Pseudo-code for IsoCon algorithm.

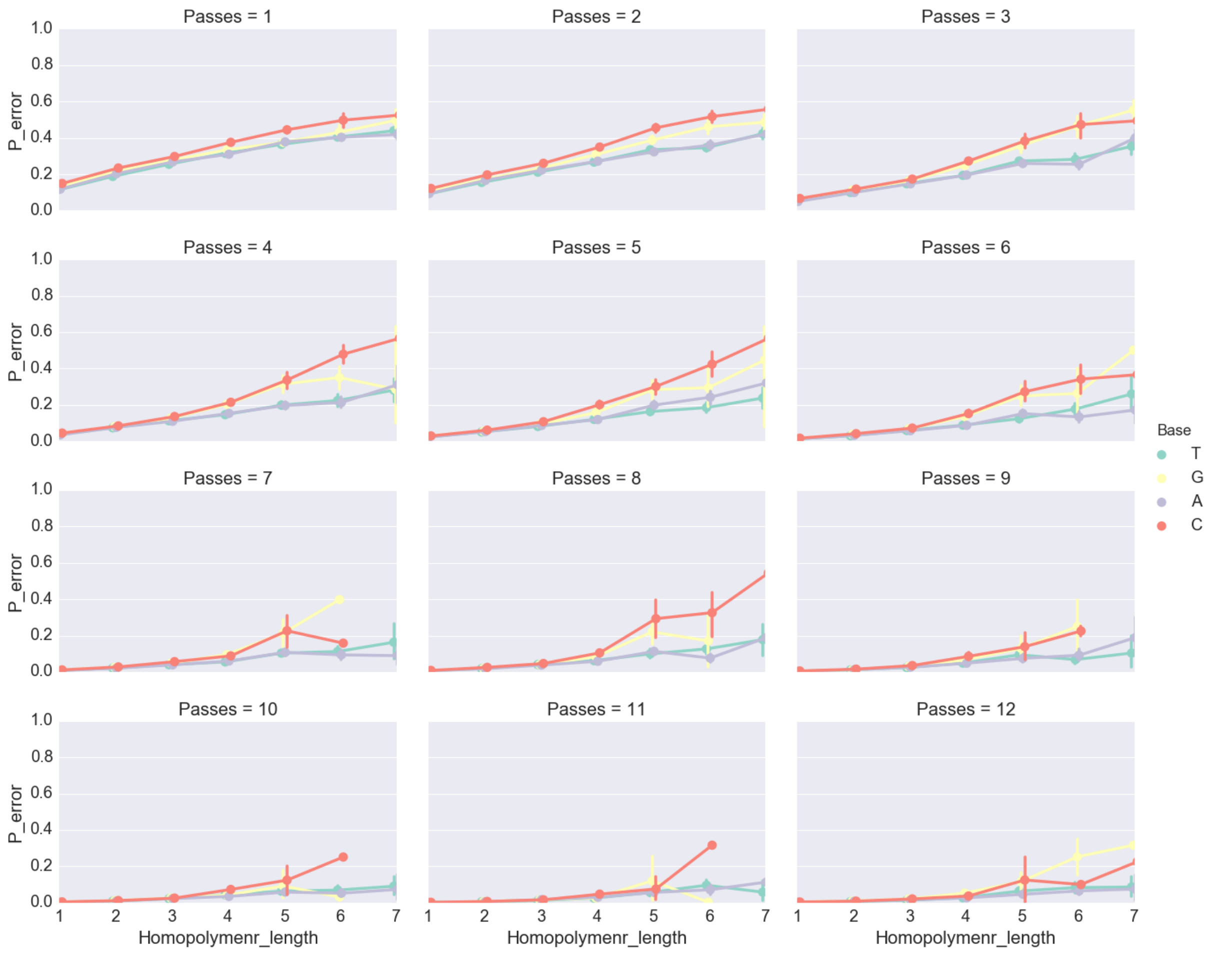

Supplementary Figure 10. Quality predictions from PacBio's base calling algorithm Arrow [21] for CCS reads based on a subsample of 5,000 CCS reads. Each panel shows Arrow's prediction that the called base is wrong (y-axis) as a function of the homopolymer length in a CCS read (x-axis). The predictions are split by the four different bases. Each point within a line is the mean probability of error for a given nucleotide and the length of the homopolymer that contains it. Vertical lines for each point are the 95% confidence interval of the mean. For each point, the number of replicates depends on the number of times the homopolymer is present in the reads. Each of the panels correspond to the shown number of passes over the read.

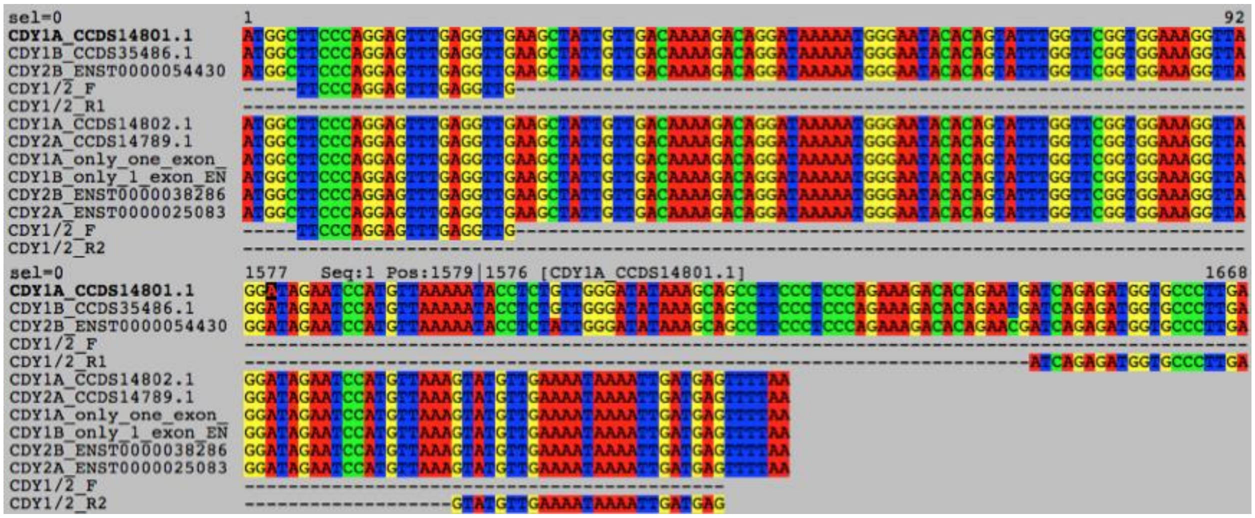

Supplementary Figure 11. Alignment of two *CDY* primer pairs (CDY1/2_F and CDY1/2_R1;CDY1/2_F and CDY1/2_R2) to transcripts from Ensembl database showing the necessity of designing two alternative reverse primers to capture all protein-coding transcripts for this ampliconic gene family.

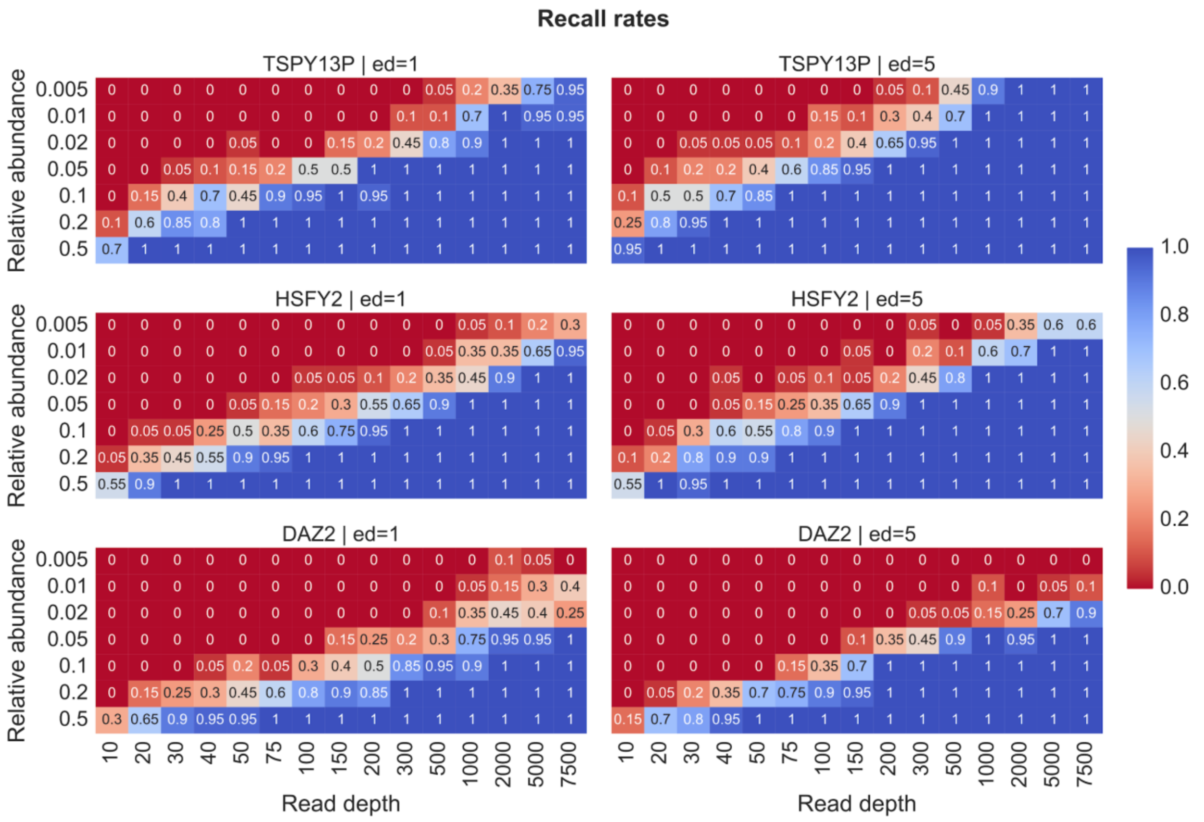

Panel A

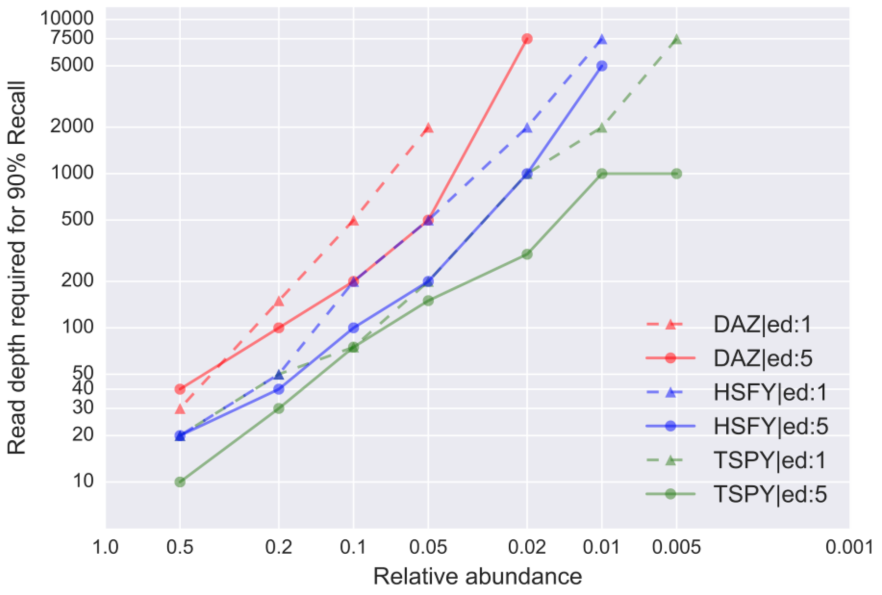

Panel B

Supplementary Figure 12. Recall power of IsoCon in controlled simulation experiments. We simulated 20 replicate experiments for each combination of given parameters: gene, edit distance, relative abundance, and number of reads. For each replicate experiment, a new target transcript is generated with a fixed number of random mutations relative to the template transcript, and reads are drawn randomly from the two transcripts. The probability of simulating a read from the target transcript is given by the relative abundance. Panel A shows the fraction of the 20 replicates where the target transcript was recovered by IsoCon. Panel B plots the minimum read depth at which a recall of >90% was achieved. Both the x- and y-axis are log-scaled. In some cases, the largest simulated sequencing depth was not enough to achieve 90% recall and no points are plotted.

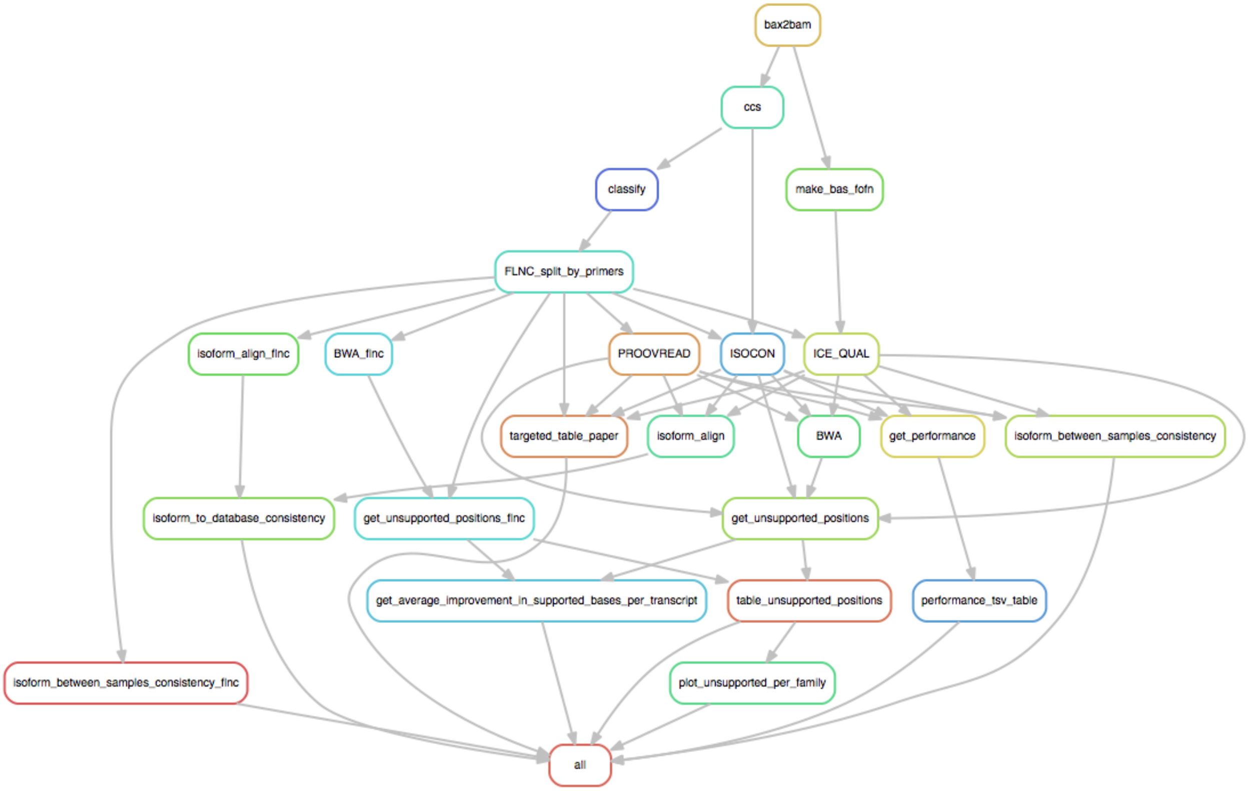

Supplementary Figure 13. Biological data processing workflow with snakemake. bax2bam, ccs, and classify are tools included in the PacBio smrtlink v4.0 tool suite. The rule split_by_primers splits the reads into batches for each given primer.

Panel A

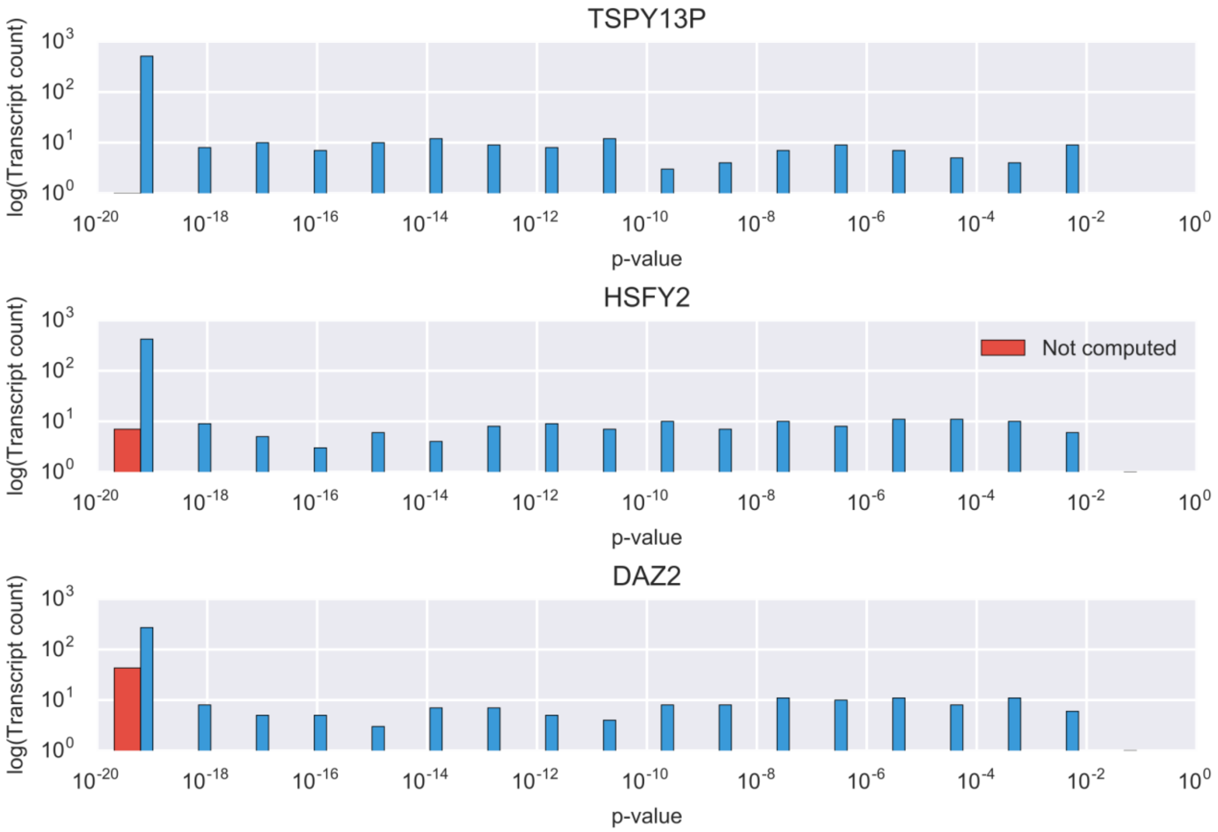

TSPY13P
log(Transcript count)
10^3
10^2
10^1
10^0
10^{-20} 10^{-18} 10^{-16} 10^{-14} 10^{-12} 10^{-10} 10^{-8} 10^{-6} 10^{-4} 10^{-2} 10^0
p-value
HSFY2
Not computed
log(Transcript count)
p-value
DAZ2
log(Transcript count)
p-value

Panel B

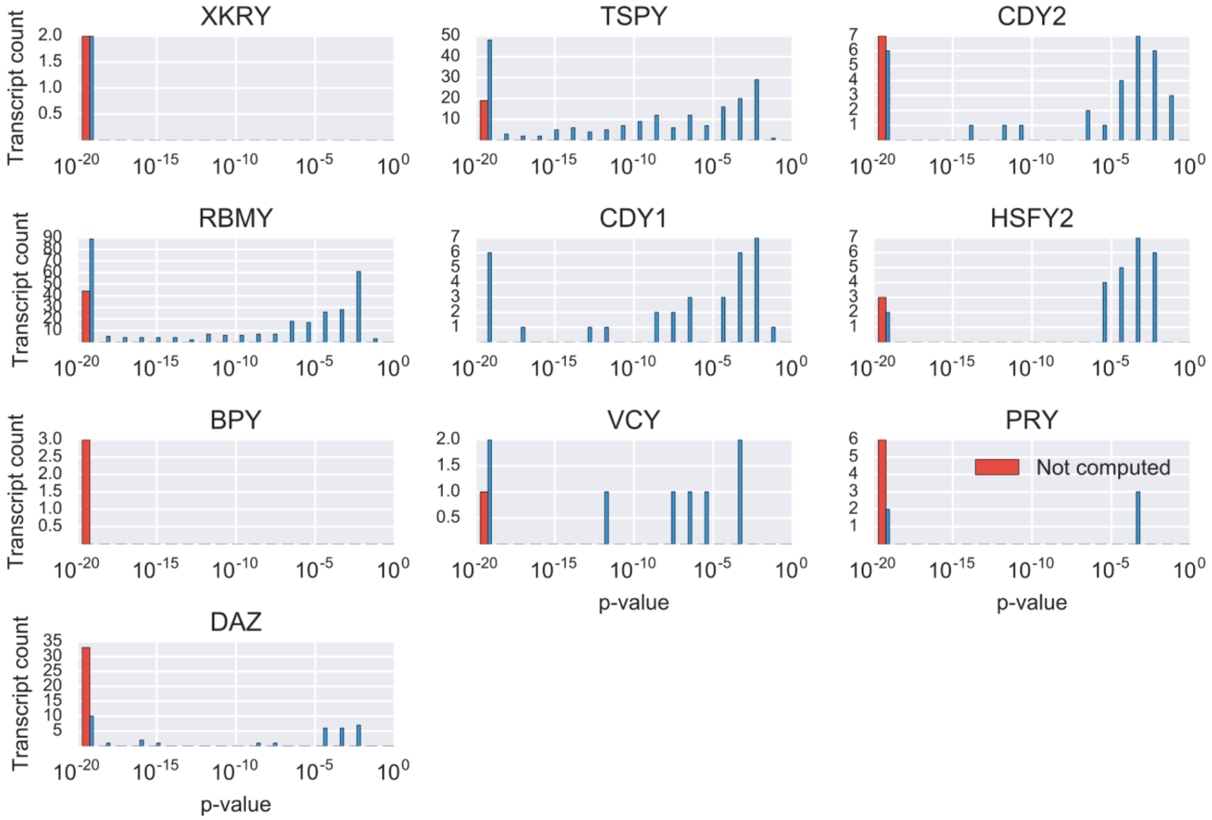

Supplementary Figure 14. P-value histograms for all predicted transcripts generated for each gene family in the simulated experiments with mutation rate 0.0001 and no exon differences **(A)** and the experimental ampliconic dataset **(B)**. For (B), the y-axis is log-scaled. Predictions with p-value lower than 10e-20 are placed in the leftmost blue bin and predictions that have more than 10 variant positions with respect to all other predictions are not statistically evaluated by IsoCon and shown in the red bin.

Panel A

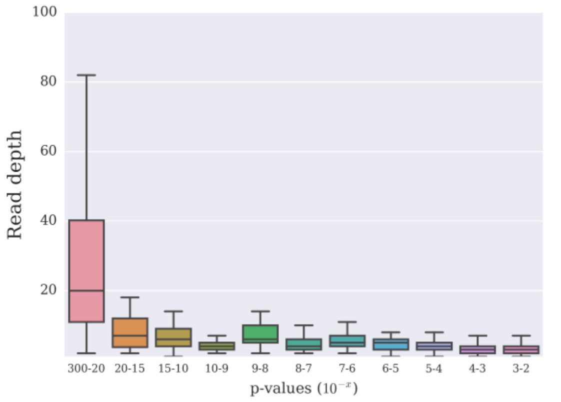

Panel B

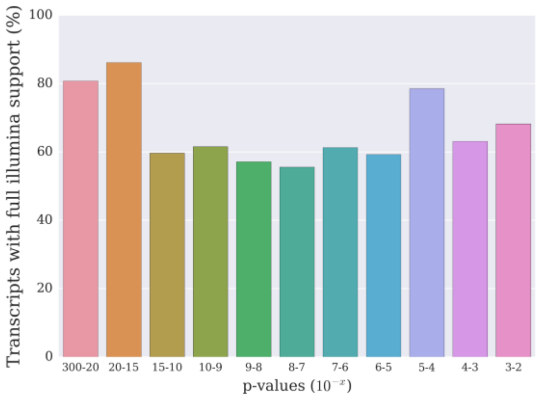

Panel C

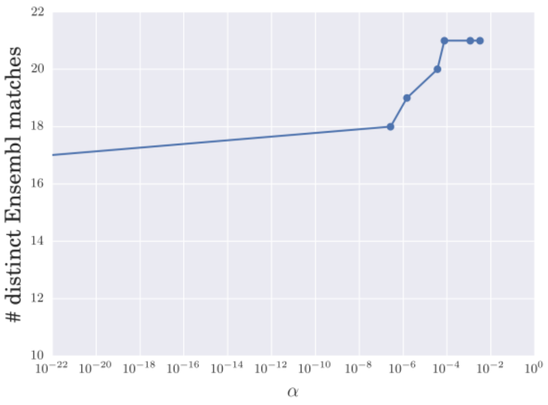

Supplementary Figure 15. Various accuracy features as a function of significance values given by IsoCon. Panel A and B shows the CCS read support and the percentage of transcripts with full Illumina support, respectively, as a function of p-value. P-values are grouped into 11 discrete ranges. Panel C shows the number of distinct Ensembl matches captured as a function of the p-value cutoff α, i.e. how many distinct Ensembl transcripts are recovered (y-axis) by transcripts with a p-value lower than x (x-axis).
