# Supplementary material for: Deciphering highly similar multigene family transcripts from Iso-Seq data with IsoCon
Source: Nat Commun. 2018 Nov 2;9:4601. doi: 10.1038/s41467-018-06910-x (PMC6214943; doi:10.1038/s41467-018-06910-x)
Supplement: Supplementary file 2 — Description of Additional Supplementary Files [file 41467_2018_6910_MOESM2_ESM.pdf]

PennState

Paul Medvedev
Assistant Professor
Department of Computer Science and Engineering
Department of Biochemistry and Molecular Biology
The Pennsylvania State University

506B Wartik Lab
University Park, PA 16802-5807
pzm11@psu.edu
814-865-0193

LEGENDS FOR SUPPLEMENTARY DATA FILES

Supplementary Dataset 1. Splice coordinates (chrY) of the 668 alignments from the 168 known database transcripts used as a database of known sites. Each row corresponds to a distinct alignment, and transcripts with multiple alignments have multiple rows. An empty splice coordinate column means that the transcript had no splice junction.

Supplementary Dataset 2. Sequences of IsoCon transcripts, their alignments, and the gene family graphs.
