# Supplementary figures and images for: Deciphering highly similar multigene family transcripts from Iso-Seq data with IsoCon
Source: Nat Commun. 2018 Nov 2;9:4601. doi: 10.1038/s41467-018-06910-x (PMC6214943; doi:10.1038/s41467-018-06910-x)

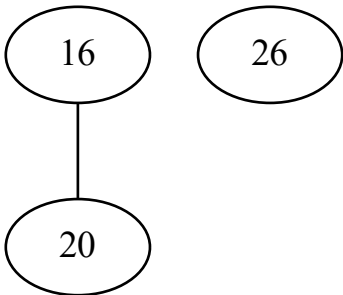

Supplement: Supplementary file 4 — Supplementary Data 2 [file 41467_2018_6910_MOESM4_ESM.zip › graphs/CDY2.pdf]

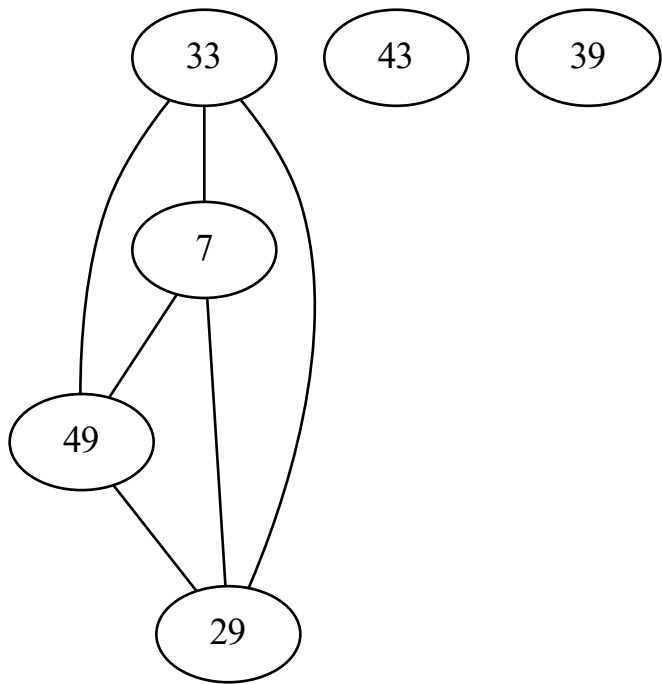

Supplement: Supplementary file 4 — Supplementary Data 2 [file 41467_2018_6910_MOESM4_ESM.zip › graphs/DAZ.pdf]

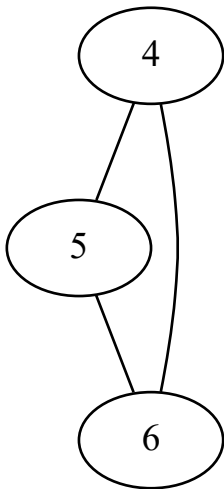

Supplement: Supplementary file 4 — Supplementary Data 2 [file 41467_2018_6910_MOESM4_ESM.zip › graphs/PRY.pdf]

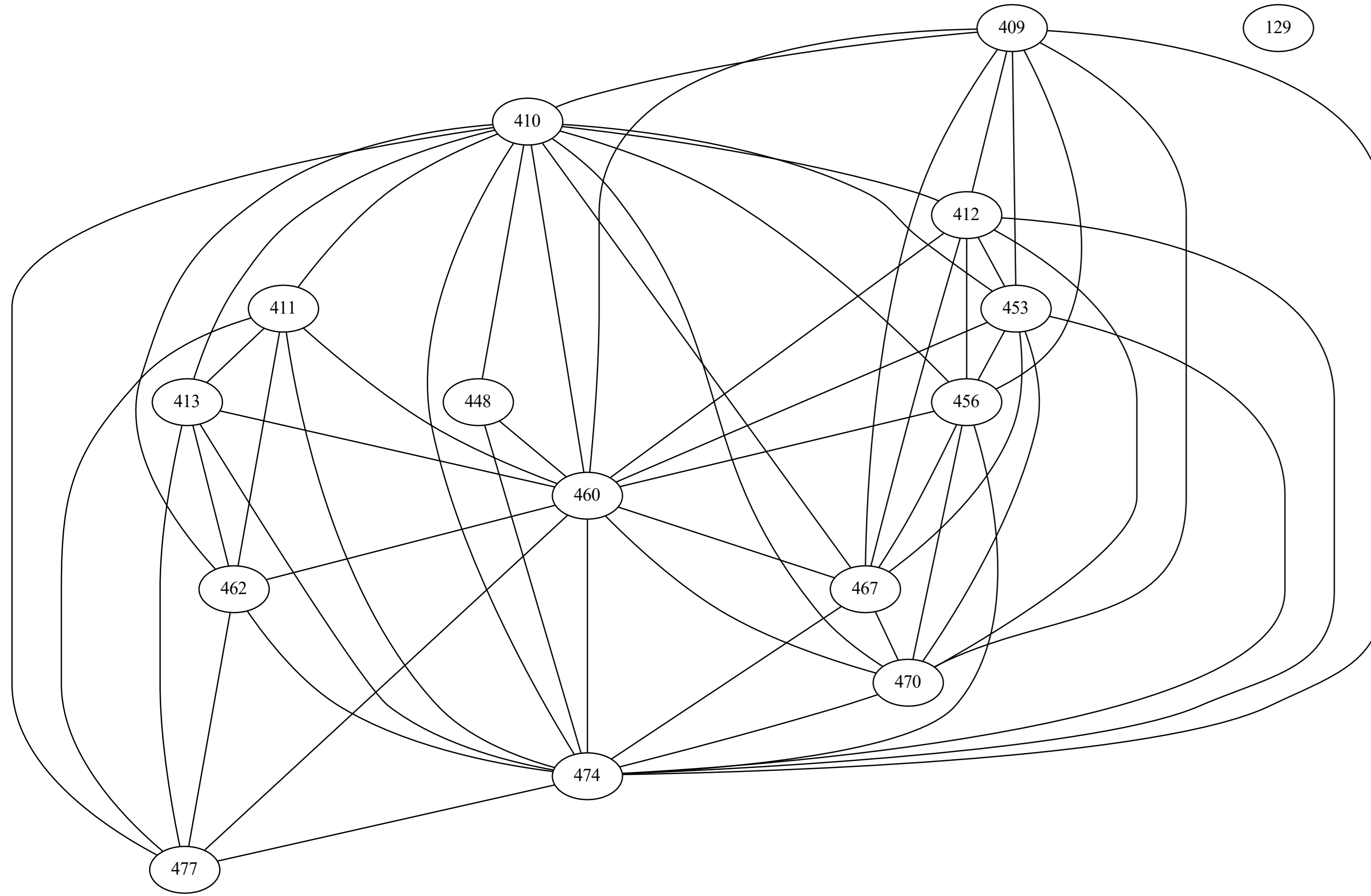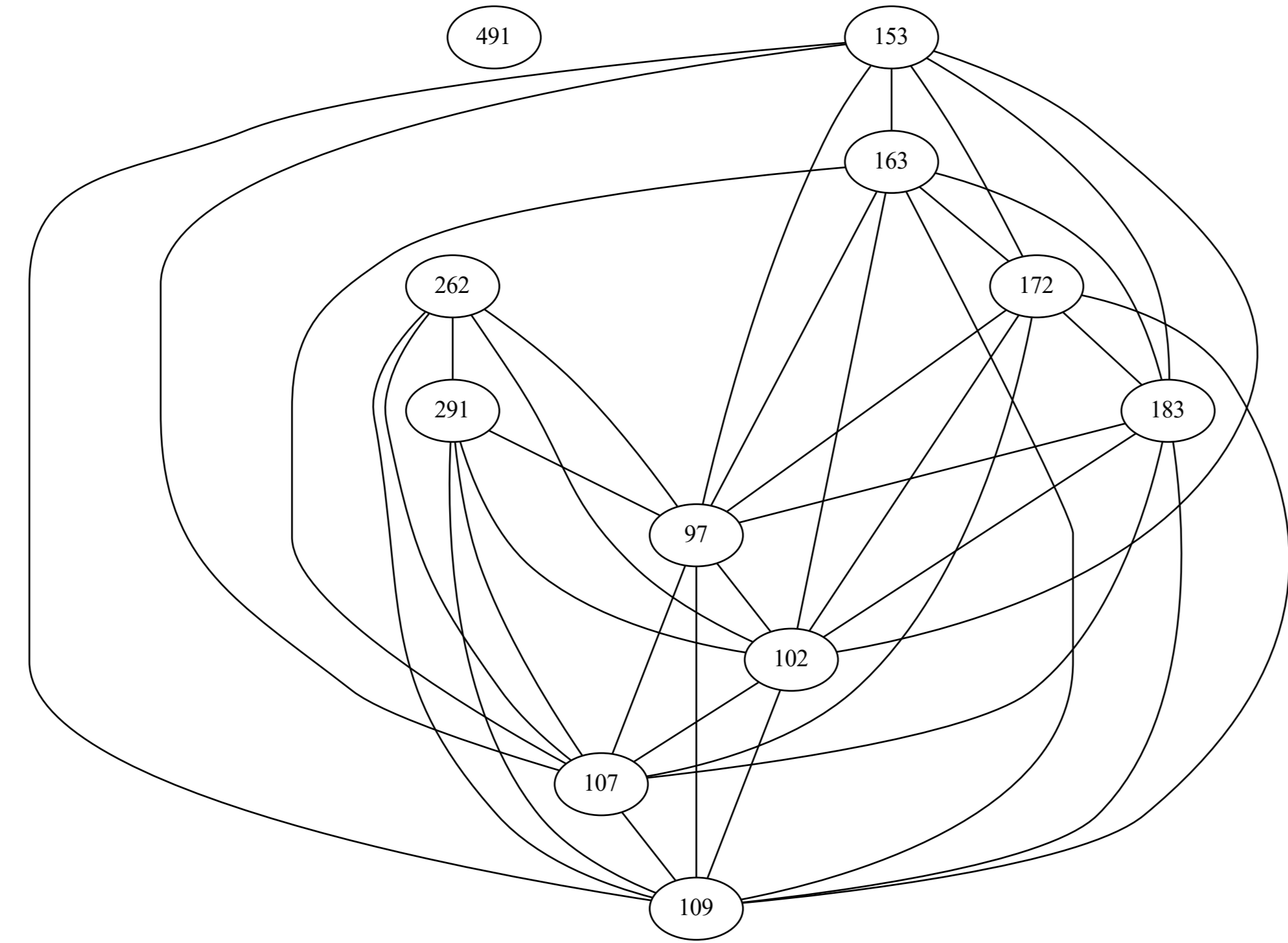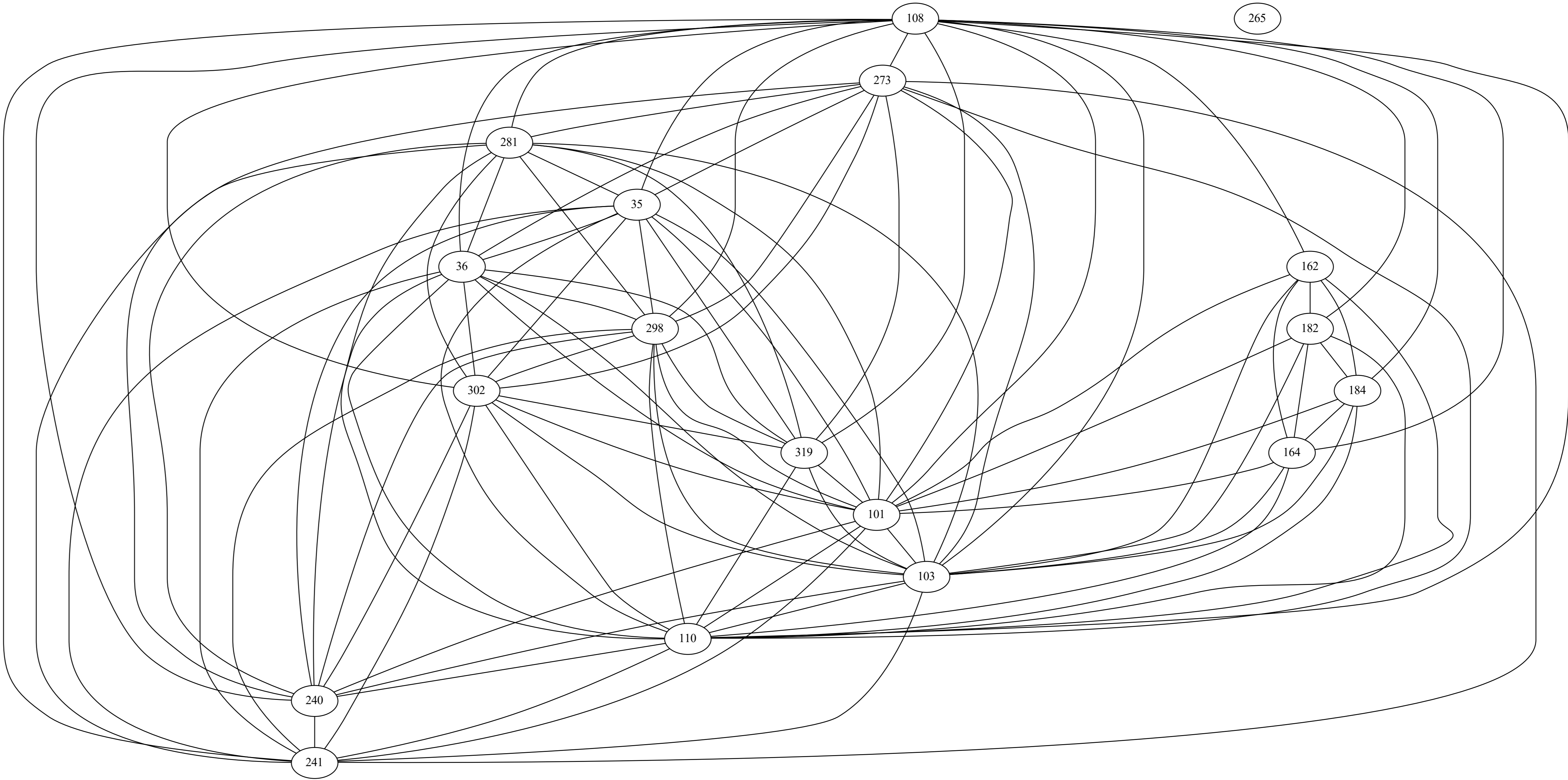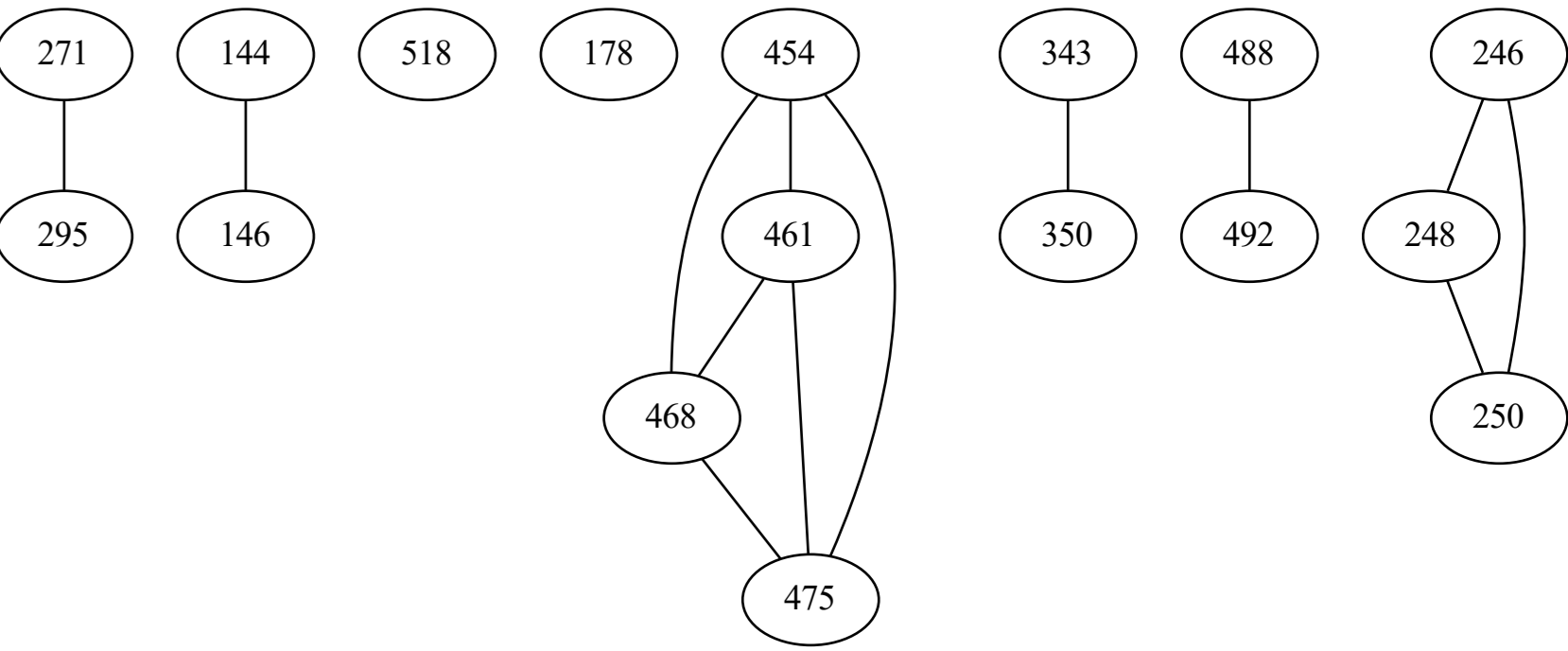

Supplement: Supplementary file 4 — Supplementary Data 2 [file 41467_2018_6910_MOESM4_ESM.zip › graphs/RBMY.pdf]

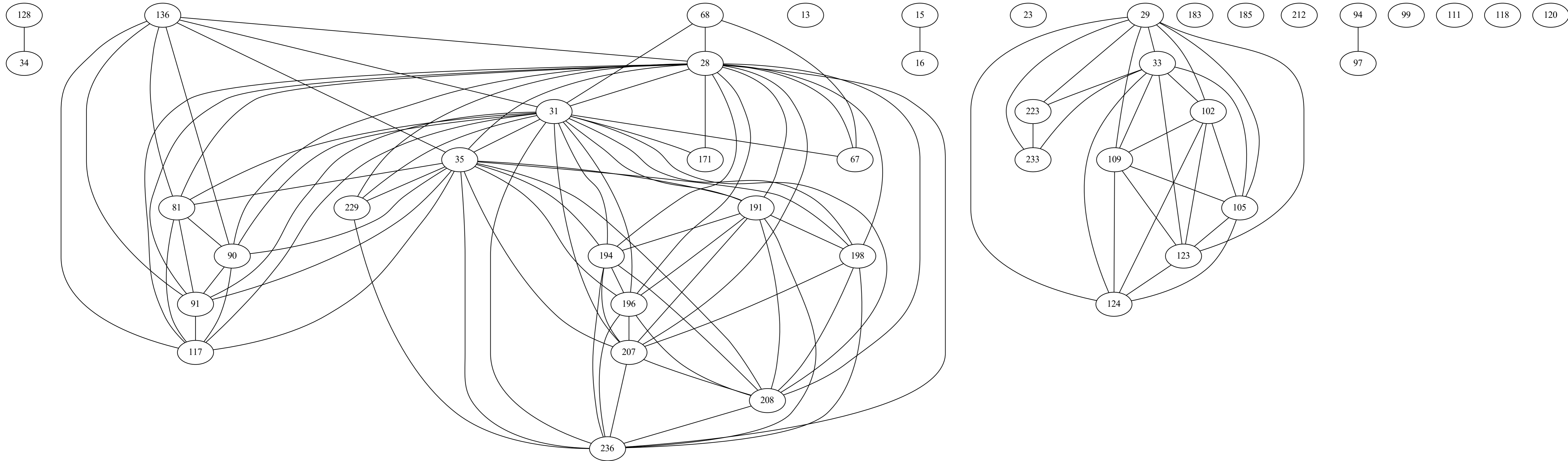

Supplement: Supplementary file 4 — Supplementary Data 2 [file 41467_2018_6910_MOESM4_ESM.zip › graphs/TSPY.pdf]

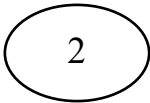

Supplement: Supplementary file 4 — Supplementary Data 2 [file 41467_2018_6910_MOESM4_ESM.zip › graphs/XKRY.pdf]

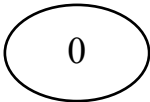

Supplement: Supplementary file 4 — Supplementary Data 2 [file 41467_2018_6910_MOESM4_ESM.zip › graphs/BPY.pdf]

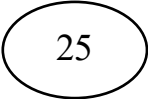

25

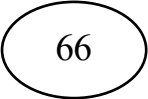

66

Supplement: Supplementary file 4 — Supplementary Data 2 [file 41467_2018_6910_MOESM4_ESM.zip › graphs/CDY1.pdf]
